# The adenovirus E4orf1 protein initiates a feedback loop involving insulin and growth factor receptors, AKT, and NF-κB, leading to abnormal DNA content in infected cells

**DOI:** 10.1371/journal.ppat.1013202

**Published:** 2025-10-27

**Authors:** Madison Moore, Jiang Kong, Ahlam Akmel, Michael A. Thomas

**Affiliations:** 1 Department of Biology, College of Arts and Sciences, Howard University, Washington, D.C., United States of America; 2 Department of Microbiology, School of Medicine, Howard University, Washington, D.C., United States of America; State University of New York Upstate Medical University, UNITED STATES OF AMERICA

## Abstract

Abnormal DNA levels, such as aneuploidy and polyploidy, can indicate cellular transformation and cancer; however, the mechanisms remain poorly understood. All tumor viruses inherently cause abnormal DNA content in cells due to their oncogenes. During infections, adenovirus (Ad) oncogenes—early region 1A (E1A), early region 4 open reading frame 3 (E4orf3), and E4 open reading frame 1 (E4orf1)—promote the abnormal buildup of cellular DNA. Previous studies have described how E1A and E4orf3 lead infected cells to accumulate abnormal DNA content; however, the role of E4orf1 remains speculative. In this study, we generated cells that express E4orf1 to investigate its role in abnormal DNA content. The E4orf1-expressing cells initially exhibited no increase in DNA content compared to the control group. However, after Ad infection, they displayed higher ploidy levels. To detail how E4orf1 influences ploidy levels in Ad-infected cells, we employed pharmacological agents that target E4orf1 signaling. Our results indicate that E4orf1 enhances signaling from insulin and growth factor receptors to AKT and NF-κB, creating a feedback loop that elevates levels of cellular DNA in Ad-infected cells.

## Author summary

The early region 4 open reading frame 1 of adenovirus (E4orf1) is recognized for its ability to initiate signals that convert normal cells into cancerous ones. During the early stages of cancer, cells show DNA content higher than the typical levels seen in the G2 and M phases of the cell cycle. This study shows that E4orf1 can trigger a feedback loop involving EGFR, INSR, IGF1R, Akt, and NF-κB, which depends on and also operates independently of PI3K, leading to the accumulation of abnormal DNA content.

## Introduction

During the early stages of cancer development, some cells increase their genomic content beyond 4N, which is found in G2 and M phase cells [1–4]. The factors that drive this increase in DNA content remain unclear. When injected into newborn rodents, adenovirus (Ad) induces malignant tumors [5]. Like other tumor viruses [6–9], it also causes abnormal cellular DNA content, such as aneuploidy and polyploidy [9]. The main contributors, the Ad oncogenes, include early region 1A (E1A), early region 4 (E4) open reading frame 3 (E4orf3), and early region 4 open reading frame 1 (E4orf1). Previous studies describe how E1A [10] and E4orf3 [11] trigger abnormal DNA levels in cells. However, the mechanism by which E4orf1 contributes to this process has yet to be elucidated.

E4orf1 is an adapter molecule that contains a domain 2 (D2) and a PDZ (Postsynaptic density protein of 95 kDa (PSD95), Drosophila disc large (Dlg), and Zonula occludens-1 (Zo-1)) protein domain-binding motif (PBM) [12]. These features enable E4orf1 to interact with the epidermal growth factor receptor (EGFR), the insulin receptor (INSR), and the insulin-like growth factor 1 receptor (IGF1R) [12] and with phosphatidylinositol 3-kinase (PI3K) [13]. In doing so, E4orf1 regulates mitogen-activated protein kinase/extracellular signal-regulated kinase (MAPK/ERK) signaling and further activates the PI3K/Akt/mTOR pathways [12–14]. The signals mediated by E4orf1 are conserved among human Ads [15,16], allowing infected cells to survive [17]. In this study, we investigated the role of E4orf1 in the abnormal DNA content observed in cells infected with Ad. Our research indicates that E4orf1 signals from receptor tyrosine kinases (RTKs)—including EGFR, INSR, and IGF1R—initiate a feedback loop involving Akt and NF-κB, resulting in abnormal DNA content in cells infected with Ad. This feedback loop may be crucial to how E4orf1 converts normal cells into cancer.

## Results

### Effects of E4orf1 on abnormal DNA content in cancer and near-normal cells

In S1A Fig, we identified the various stages of the cell cycle by analyzing DNA content with propidium iodide (PI) [20,21]. As previously [18,19], we excluded doublets and clumps that could skew the data by first gating the cells (S1Ai Fig). Most of the cells are in the G1 phase (S1Aii and S1Aiii Fig). Cells with fragmented DNA, dead cells, have a DNA content less than 2N (DNA content <G1 in S1Aii Fig), which is the typical amount found in G1 phase cells. During the S phase, DNA synthesis increases the DNA content to 4N, seen in G2 phase cells. The G2 cells then proceed to the M phase, where they undergo cell division. Some cells fail to divide and accumulate a DNA content greater than that of G2/M phase cells (DNA > 4N in S1Aii Fig). Using this approach, far more cells infected with Ad show abnormal DNA content compared to the mock-infected control cells (S1Aii-S1Avi Fig).

A complete list of the viruses used in this study is included in Table 1 of the Materials and Methods section. Similar to other tumor viruses [7], replication-defective Ads, such as the *E1B55K*-deleted *dl*1520 [20] and *E4orf6*-deleted *dl*355* [21] in S1C Fig, induced levels of abnormal DNA content in cells that greatly exceeded those of *dl*309, an otherwise phenotypic wild-type Ad [22,23] (S1B Fig). We also noticed that as long as the Ads include E1B55K and E4orf6 (shown in S1B Fig by *in*351 and elsewhere with other Ads [24]), the inactivation of *E4orf1* does not affect levels of abnormal DNA content. Therefore, we deleted the *E4orf1* gene from the *E1B55K*-deleted Ad [25], referring to this *E1B55K-* and *E4orf1-*deleted Ad as *E4orf1(****-****)*. We chose the E1B55K-deleted Ad over the E4orf6-deleted Ad because we had previously demonstrated that the E1B55K-deleted Ad may have vaccine potential, allowing us to learn more about it. In all experiments, more **E4orf1(*+)* Ad-infected cells (Table 1) accumulated abnormal DNA content than *E4orf1(****-****)* Ad-infected cells (S1Aiv-S1Av Fig) [11]. Thus, E4orf1 is associated with enhanced levels of abnormal DNA content in Ad-infected cells.

**Table 1:** List of viruses referenced throughout the text.

Viruses	Major changes	Previously	1^st^ Referenced
*E4orf1(+)*	*E3-deleted*	*dl309, ∆E3*	(*Jones & Shenk, 1979*)
*E4orf1(-)*	*E4orf1*-deleted	*in351, ∆E4orf1*	(*Halbert et al, 1985*)
*E4orf1(+)*	*E1B55K*-deleted	*dl1520, ∆E1B55K*	*(Barker & Berk, 1987)*
*E4orf1(+)*	*E4orf6*-deleted	dl355*, ∆E4orf6	*(Huang, & Hearing 1989)*
*E4orf1(+)*	*E2B*- and *E3*-deleted	*MAd5rFLSC, ∆E3*	*(Thomas et al, 2013)*
*E4orf1(-)*	*E1B55K*-, and *E4orf1*-deleted	*∆E1B/∆E4orf1, ∆E4* ^1^	(*Sangare et al, 2022*)
*E4orf1(-)*	*E1B55K*-, and *E4orf1*-E4orf3-deleted	*dl1016, ∆E1B55K/∆E4orf1–3*	(*Bridge, & Ketner, 1990*)

**Fig 1:**
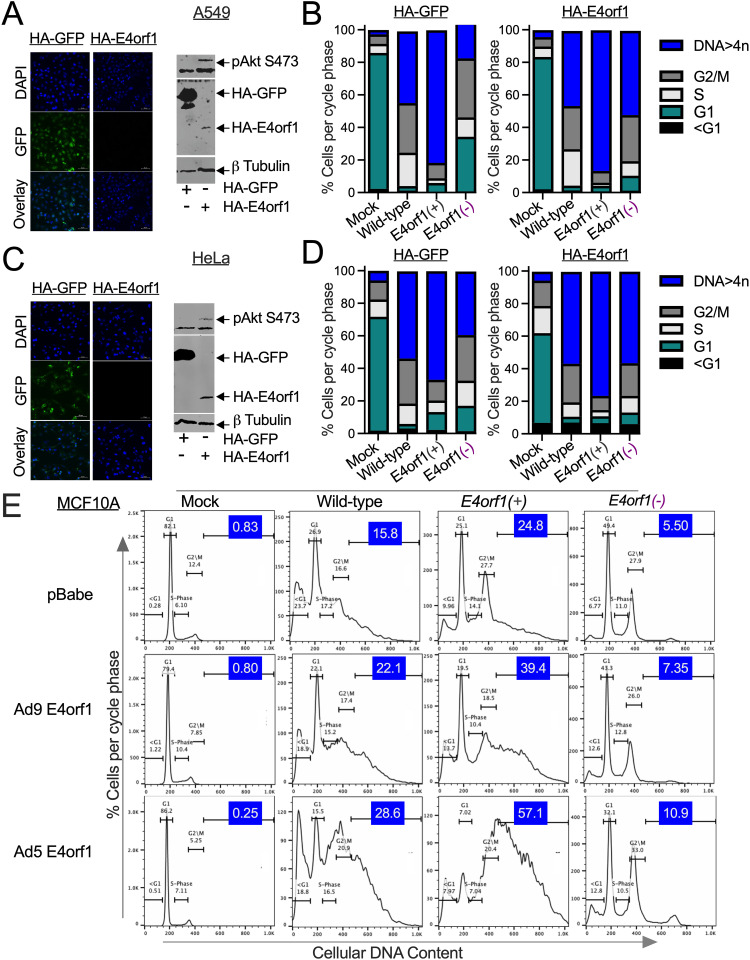
Effects of E4orf1 on the number of cancer and near-normal cells that accumulate abnormal DNA content. (A, B) A549 and (C, D) HeLa cells were transfected with MSCVN-HA-GFP or MSCVN-HA-E4orf1 plasmids [29]. The transfected cells were cultured in selection media for 7 to 10 days. (A, C) The cells were photographed at 20X magnification with a spinning disk confocal microscope to confirm the expression of the indicated plasmids. In these images, DAPI stains the nuclei of the cells. (A, C) An immunoblot was used to distinguish the HA-tagged GFP from the HA-tagged E4orf1. Phosphorylated Akt S473 and the β-tubulin loading control are also shown. (B, D) The HA-tagged GFP-expressing cells and the HA-tagged E4orf1-expressing cells were infected at an MOI of 25 pfu/cell with the indicated Ads for 48 hours. For each group of (B) A549 and (D) HeLa cells, the percentage of cells in each cell cycle phase (S1 Table) was plotted in GraphPad Prism and is shown. The data shown is representative of one of at least three experiments. (E) Empty pBabe vector-, Ad9 E4orf1- and Ad5 E4orf1-expressing MCF10A cells were infected with the above-listed Ads and incubated for 48 hours. The *E4orf1(-)* Ad here, *dl*1016 contains the deletion E1B55K, E4orf1, E4orf2 and E4orf3 [30]. Examples of the DNA cell cycle profiles are displayed. The fraction of cells with abnormal DNA content is highlighted in blue. The data shown is representative of one of at least three experiments.

To confidently declare E4orf1’s role in the abnormal DNA content, we generated GFP- and E4orf1-expressing A549 and HeLa cells (Fig 1A–1D). Immunofluorescence microscopy revealed that the negative control cells expressed GFP, while E4orf1-expressing cells did not (Fig 1A and 1C). The HA-tagged GFP (30Kda) and E4orf1 (19Kda) are further distinguished by size using immunoblotting (Fig 1A and 1C). Of the two cell lines, Akt, the primary target of E4orf1 [13,26], was observed phosphorylated only in the E4orf1-expressing cells (pAkt S473 in Fig 1A and 1C). Flow cytometry was used to interrogate the cell cycle DNA profiles of the cells. The mock-infected GFP and E4orf1-expressing cells displayed similarly low levels of abnormal DNA content (Fig 1B and 1D).

In rodents infected with Ad subtype 9 (Ad9), E4orf1 is solely responsible for tumor development [27]. Although less effective, the E4orf1 from Ad type 5 (Ad5), used in this study, also activates the PI3K pathway and induces cell transformation [16]. Demonstrating that E4orf1 from Ad9 acts similarly to E4orf1 from Ad5 in influencing DNA content would extend our findings to these Ad, where E4orf1 is the oncogenic determinant. MCF10A is a non-tumorigenic breast epithelial cell line, serving as a dependable model for normal human breast cells [28]. Therefore, we repeated the experiments from parts B and D above in MCF10A cells that stably express either an empty retroviral vector (pBabe) or pBabe containing Ad9 E4orf1 or Ad5 E4orf1 [13,16]. Examples of the DNA cell cycle profiles are shown in Fig 1E, with the percentage of cells exhibiting abnormal DNA content highlighted in blue. Similar to the A549 and HeLa cells, only a small percentage of the mock-infected, Ad9, and Ad5 E4orf1-expressing MCF10A cells displayed abnormal DNA content (see the first column of Fig 1E). Therefore, E4orf1 expression in both cancerous and non-cancerous cells does not lead to abnormal DNA content.

We then examined whether expressing E4orf1 in cells could increase the percentage of Ad-infected cells that develop abnormal DNA content. More E4orf1(-) Ad-infected E4orf1-expressing A549 and HeLa cells develop abnormal DNA content compared to infected GFP-expressing cells (Fig 1B and 1D). In the MCF10A cells, both E4orf1 from Ad9 and from Ad5 increase the percentage of Ad-infected cells with abnormal DNA content compared to the mock-infected cells (Fig 1E, compare the 2nd and 3rd rows, and the 2nd, 3rd, and 4th columns to the columns in the 1st row). Thus, unlike when E4orf1 is expressed alone, it enhances the levels of abnormal DNA content that accumulate in both cancerous and near-normal Ad-infected cells.

### Effects of serum depletion on the number of Ad-infected cells with abnormal DNA content

**Fig 2:**
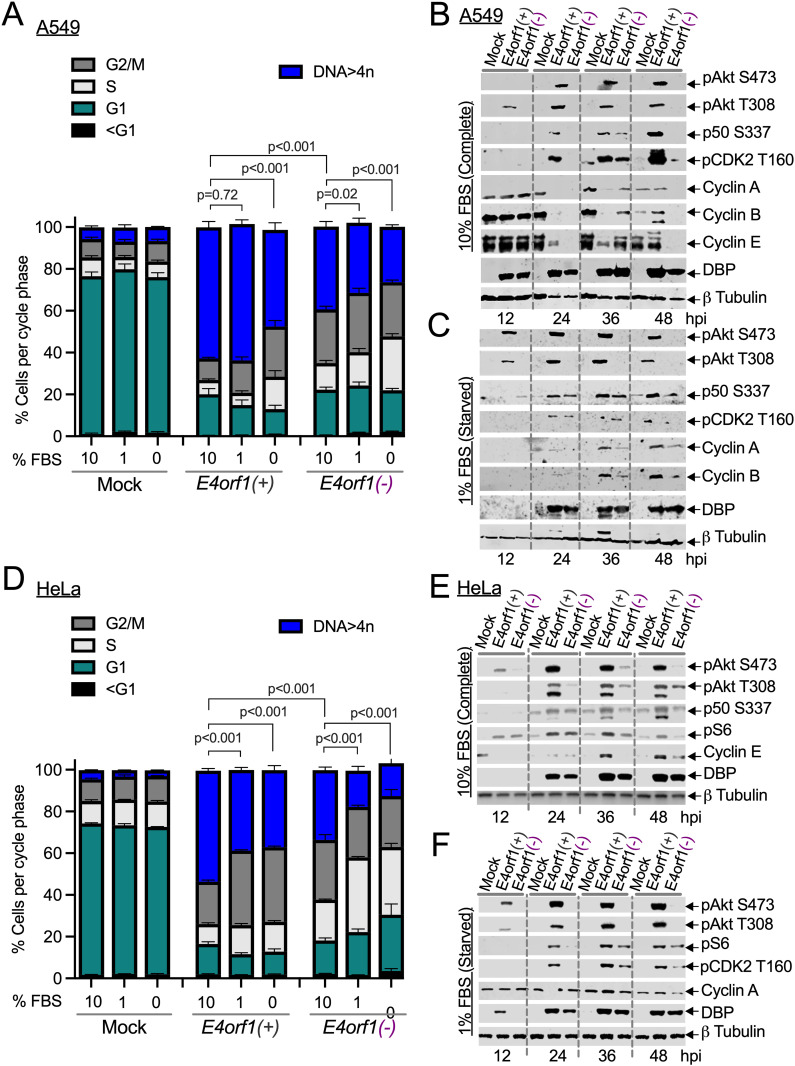
Effects of serum levels on abnormal DNA content in Ad-infected cells. (A) Groups of A549 (n = 4-11) and (D) HeLa cells (n = 4-6) were infected at an MOI of 25 pfu/cell with the *E4orf1(+)* or the *E4orf1(-)* Ad and incubated in media containing 10%, 1%, or 0% fetal bovine serum (FBS) for 48 hours. The cells were analyzed by flow cytometry. The average percentages of cells in each cell cycle phase (S3 Table), along with their individual SEM, are shown in the graphs. P values were determined using a two-way ANOVA with Holm–Šídák’s multiple comparisons test. A full set of statistics is available in S3 Fig. (B, C) A549 and (E, F) HeLa cells were infected with the indicated Ads and incubated for 12, 24, 36, or 48 hours in (B, E) 10% or (C, F) 1% FBS. The lysed cells were analyzed using immunoblotting to detect one or more of the following: phosphorylated Akt serine 473 (pAkt S473), pAkt T308, NF-κB p50 S337 (p50 S337), pS6 S235/S236 (pS6), pCDK2 T160, cyclins A, B, and E, DBP (Ad infection control), and β-tubulin (loading control). The data shown represents one of at least three experiments conducted per cell under each condition.

E4orf1 has been reported to promote glucose uptake and increase metabolism in serum-rich and serum-starved environments [31–34]. Therefore, we investigated whether serum levels influenced the ability of Ad to promote abnormal DNA content in cells (Fig 2A and 2D). More **E4orf1(*+)* Ad-infected cells acquired abnormal DNA content than *E4orf1(****-****)* Ad-infected cells at every serum level evaluated (Fig 2A and 2D). Since lowering the serum levels ultimately reduced the number of cells with abnormal DNA content (Fig 2A and 2D), we conclude that extracellular factors contribute to the propensity for polyploidization in Ad-infected cells.

Akt [35,36], NF-κB [37,38], the cyclins, and CDK2 [39–41] facilitate cell cycle progression and are also known to transmit insulin and growth factor signaling to control cellular metabolism [42–46]. Therefore, we assessed the status of one or more of these proteins in Ad-infected cells using immunoblots. Regardless of serum levels, a 12-hour incubation with *E4orf1(+)* Ad triggered the phosphorylation of Akt (Fig 2B, 2C, 2E and 2F). At later time points (24, 36, and 48 hpi), in addition to Akt, phosphorylated NF-κB p50, S6, and CDK2 were also detected (Fig 2B, 2C, 2E and 2F). This starkly distinguished the mock and *E4orf1(-)* Ad-infected cells from those infected with *E4orf1(+)* Ad. Moreover, pharmacological agents that inhibited Akt (AKTIV, Torin 1, LY294002) or its ability to signal to key mediators (Rapamycin) also negatively impacted the levels of abnormal DNA content (S2 and S3 Figs). Consequently, intracellular mediators involved in cell cycle progression and metabolism may affect ploidy levels in Ad-infected cells.

### Effects of Ad on the insulin-like growth factor 1 receptor

**Fig 3:**
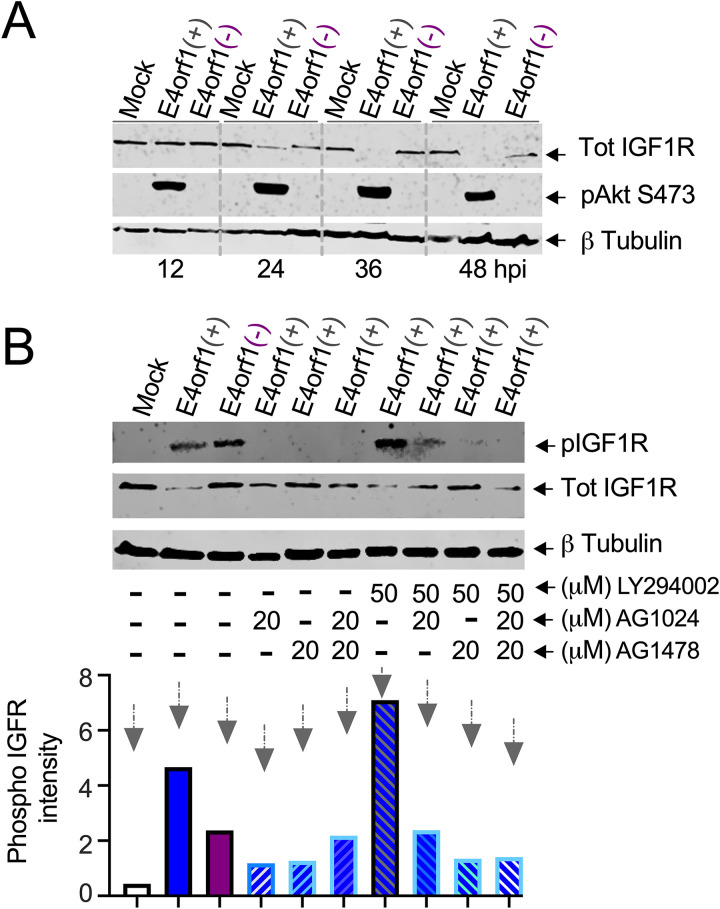
Effects of Ad on the insulin-like growth factor 1 receptors. (A) HeLa cells were infected with the indicated Ads for 12, 24, 36, and 48 hours. The cells were lysed and analyzed by immunoblotting for pAkt (S473), IGF1R, and β-tubulin. The data shown represents one of at least three experiments. (B) Hela cells were infected with the indicated Ads, and four hpi they were exposed to either AG1478, AG1024, or LY294002 and allowed to incubate in 10% FBS for 48 hours. The lysed cells were analyzed by immunoblot probing for IGF1R (Tot IGFR), pIGF1R Tyr1135/1136 (pIGF1R), and β-tubulin. The normalized pIGF1R intensity values (S4 Table) were plotted and shown below. The data shown represents one of at least three experiments conducted.

E4orf1 interacts with the epidermal growth factor (EGF) receptor (EGFR) and forms a quaternary complex with the insulin (INS) receptor (INSR) and the insulin-like growth factor 1 (IGF1) receptor (IGF1R) [12], which controls metabolism [31]. Therefore, we evaluated the status of IGF1R in Ad-infected cells grown in 10% FBS over time. At 12 hpi, although IGF1R was detected at similar levels in all the cells, only the *E4orf1(+)* Ad-infected cells showed phosphorylated Akt (Fig 3A). Noticeably, at 24 hpi, levels of IGF1R appeared somewhat reduced in **E4orf1(*+)* compared to mock and *E4orf1(****-****)* Ad-infected cells (Fig 3A). At 36 and 48 hpi, the IGF1R antibody failed to detect IGF1R in *E4orf1(+)* Ad-infected cells, while it was still detectable in mock and *E4orf1(-)* Ad-infected cells (Fig 3A).

Fig 3A indicates that the IGF1R may undergo modifications during Ad infection. Among these potential modifications, phosphorylation has been reported in other studies involving E4orf1 [12]. Phosphorylation of the IGF1R affects the receptor’s activity, downstream signaling, and cellular processes, including growth and survival [47]. As a result, we tested this in our systems. Surprisingly, the IGF1R was phosphorylated in both *E4orf1(+)* and *E4orf1(****-****)* Ad-infected cells (Fig 3B). Therefore, E4orf1 might not be the only Ad product responsible for stimulating IGF1R phosphorylation during Ad infection.

Given that the IGF1R levels were again reduced in the *E4orf1(+)* Ad-infected cells, we normalized the levels of phosphorylated IGF1R. According to the normalized values (bar graph at the bottom of Fig 3B), the IGF1R phosphorylation was approximately twice as high in *E4orf1(+)* Ad-infected cells compared to *E4orf1(-)* Ad-infected cells (Fig 3B). Exposure of the *E4orf1(+)* Ad-infected cells to either AG1478, which blocks EGFR activity, or AG1024, which blocks IGF1R activity, reduced the phosphorylation of the IGF1R (Fig 3B). These findings are consistent with previous research indicating the activation of EGFR and IGF1R in Ad-infected cells [12].

### Effects of insulin and growth factor receptors on abnormal DNA content in Ad-infected cells

**Fig 4:**
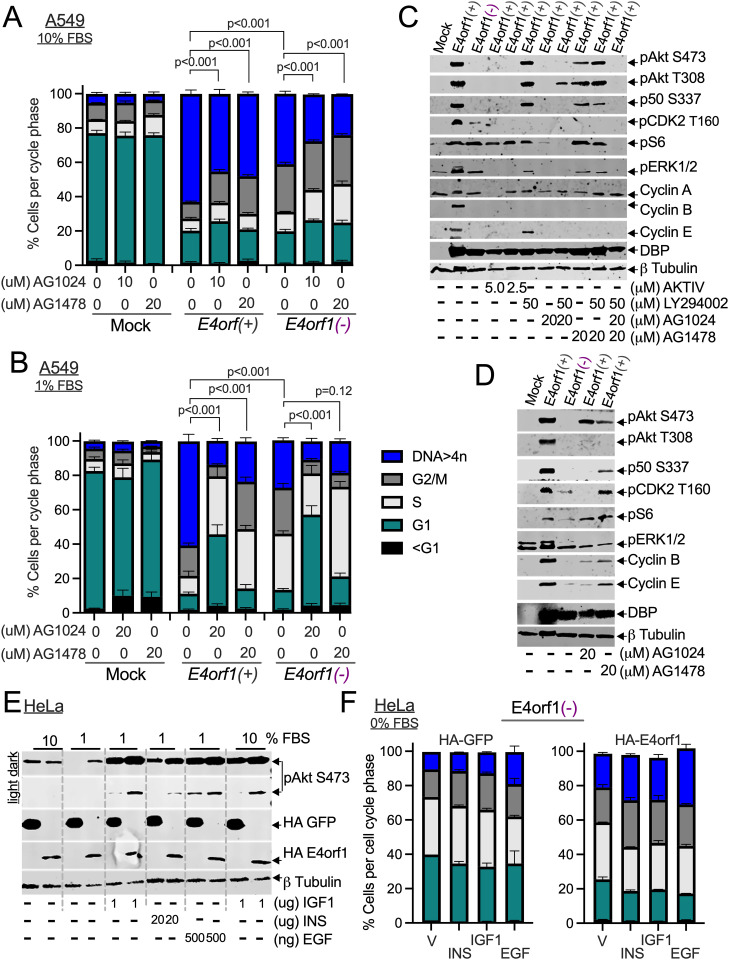
Effects of insulin and growth factor receptors on abnormal DNA content in Ad-infected cells. (A, B) A549 cells were infected with the indicated Ads, and four hpi were exposed to the specified concentrations of AG1478 or AG1024. (A) The cells were incubated in 10% or (B) 1% FBS for 48 hours. The stained cells were analyzed by flow cytometry. For (A) n = 3-6, (B) n = 5-9, and (C) n = 3-6, averages of the percentage of cells in each phase of the cell cycle (S5 Table) with their individual SEM were plotted in GraphPad Prism and shown. The P values were calculated using a two-way analysis of variance (ANOVA) with Holm–Šídák’s multiple comparisons tests. A full set of statistics is available in S6 Fig. (C, D) A549 cells were infected with the indicated Ads. Four hpi, the cells were treated with either the epithelial growth factor receptor inhibitor AG1478, the insulin-like growth factor 1 receptor inhibitor AG1024, the Akt inhibitor AKTIV, or the PI3K inhibitor LY294002 at the specified concentrations. They were then incubated in (C) 10% or (D) 1% FBS for a total of 48 hours. The lysed cells were analyzed by immunoblot to detect one or more of the following markers: pAkt (S473), pAkt (T308), NF-κB p50 (S337), pS6, pCDK2 (T160), pERK1/2, cyclin A, cyclin B, cyclin E, DBP, and β-tubulin. The data shown represents one of at least three experiments conducted. (E) HA-tagged GFP- and HA-tagged E4orf1-expressing HeLa cells were incubated in 1% FBS for 24 hours. After that, the cells were stimulated with or without 1 μg/mL IGF1, 20 μg/mL INS, or 500ng/mL EGF and incubated in either 10% or 1% FBS for 30 minutes. As described in the Method section, the lysed cells were analyzed by Western blot, probed for pAkt (S473), HA, and β-tubulin. The data shown represents one of at least three experiments conducted. (F) HeLa cells expressing HA-tagged GFP or HA-tagged E4orf1 were incubated in 0% FBS and infected with or without the *E4orf1(-)* Ad at an MOI of 25 pfu/cell. Four hpi, the cells were stimulated with 1 μg/mL IGF1, 20 μg/mL INS, or 500 ng/mL EGF. Twenty-four hours later, the stained cells were interrogated by flow cytometry. For each group (n = 2), the average percentage of cells in each cell cycle phase (S5 Table), along with their standard deviations (SD), was plotted in GraphPad Prism and is shown.

E4orf1’s interactions with EGFR, INSR, and IGF1R [12] induce metabolic changes [31] that may occur before cell transformation [48]. In Fig 3B, we confirmed the mutual dependency of IGF1R on EGFR for activation in Ad-infected cells. Therefore, we assessed how inhibiting insulin and growth factor receptor activity affects abnormal DNA content. Although a 10 μM concentration of AG1024, previously used in studies involving E4orf1 [11], effectively reduced the proportion of Ad-infected cells with abnormal DNA content (Fig 4A), these percentages fluctuated over time depending on cell number and other factors. Therefore, a higher concentration, 20 μM, also reported [46], was used throughout the study. Even though AG1024 and AG1478 effectively decreased the number of Ad-infected cells with abnormal DNA content, at 20 μM, AG1024 had a larger impact than AG1478 (Figs 4B and S5B). Nevertheless, signals from IGF1R and EGFR are crucial for Ad-infected cells to accumulate abnormal DNA content.

We investigated how inhibiting EGFR and IGF1R affects the phosphorylation of several of the proteins we previously examined, including Akt, NF-κB p50, ERK1/2, S6, and CDK2, in *E4orf1(+)* Ad-infected cells. The impact of AKTIV is shown in S2 Fig and is used here for comparison. As shown, inhibiting IGF1R was as effective at reducing the phosphorylation of Akt, NF-κB p50, ERK1/2, and CDK2 in *E4orf1(+)* Ad-infected cells as AKTIV (Fig 4C). In 10% FBS, LY294002 at the specified concentration can inhibit Akt S473 phosphorylation (S2A Fig). However, this effect is not observed in *E4orf1(+)* Ad-infected cells (Figs 4C, 4D, S2, S5C and S5D [17]). Its effectiveness, however, is reflected in the reduced phosphorylation levels of the downstream effectors CDK2 and S6 (Figs 4C, 4D, S2, S5C and S5D). Comparatively, AG1478, while not as effective as AG1024, was more effective at inhibiting the phosphorylation of Akt in 10% FBS than LY294002 (Figs 4C, 4D, S5C and S5D). In 1% FBS, all inhibitors notably reduced the phosphorylation of Akt, ERK1/2, and NF-κB p50 when applied (Figs 4D and S5D). Thus, insulin and growth factor receptors play a vital role in the phosphorylation of Akt, ERK1/2, NF-κB p50, S6, and CDK2 in *E4orf1(+)* Ad-infected cells.

Insulin and growth factor receptor activation is caused by ligand binding [49]. Consequently, we evaluated the impact of each ligand on the phosphorylation of Akt in GFP- and E4orf1-expressing cells. The GFP and E4orf1-expressing HeLa cells incubated in 10% FBS exhibited phosphorylated Akt (Fig 4E, 1^st^ two columns). By contrast, when the cells were grown in 1% FBS, only the E4orf1-expressing cells exhibited phosphorylated Akt (Fig 4E, compare 2^nd^ two columns). Following ligand stimulation, all the cells exhibited increased levels of phosphorylated Akt (Fig 4E, see the 3^rd^, 4^th^, 5^th,^ and last two columns). Nonetheless, examining shorter exposure times indicated that Akt exhibited a higher phosphorylation level in cells expressing E4orf1 (see light exposure in Fig 4E, 3rd, 4th, 5th, and last two columns).

Next, we evaluated the individual ligands’ impact on the proportion of Ad-infected cells accumulating abnormal DNA content. The mock-infected GFP- and E4orf1-expressing cells did not show any accumulation of abnormal DNA content, regardless of ligand stimulation (S5E Fig). Compared to infected GFP-expressing cells, the cells expressing E4orf1 accumulated higher levels of abnormal DNA content (Figs 4F and S5E), and this accumulation increased further after ligand stimulation (Figs 4F and S5E). In low FBS, E4orf1 induces PI3K-dependent phosphorylation of Akt (S2F Fig). When insulin and growth factor receptors are activated by their ligands, this leads to elevated levels of phosphorylated Akt, indicating that E4orf1-expressing cells respond more effectively to insulin and growth factor receptor signals. The greater the increase in Akt phosphorylation/activation levels, the more Ad-infected cells exhibit abnormal DNA content.

### Effects of NF-κB on the IGF1R and Akt in Ad-infected cells

**Fig 5:**
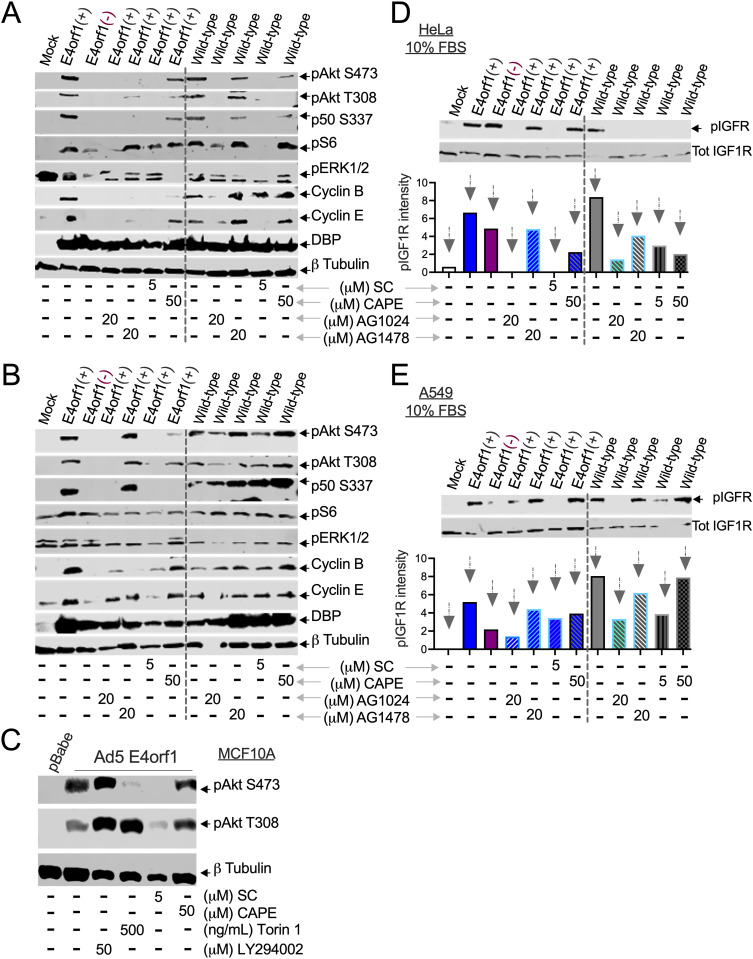
NF-κB mediates the phosphorylation of the IGF1R and Akt in Ad-infected cells. (A) HeLa and (B) A549 cells were infected with the indicated viruses, and four hpi were exposed to AG1024, AG1478, an NF-κB nuclear translocation inhibitor, caffeic acid phenethyl ester (CAPE) [51], or the NF-κB transcriptional inhibitor, SC75741 (SC) [52], at the indicated concentrations for 48 hours. The lysed cells were then analyzed by immunoblot to detect one or more of the following markers: pAkt (S473), pAkt (T308), NF-κB p50 (S337), pS6 (S235/S236), pCDK2 (T160), pERK1/2 (T202/Y204), cyclin B, cyclin E, DBP, and β-tubulin. The data shown represents one of at least three experiments conducted. (C) Empty pBabe vector- and Ad5 E4orf1-expressing MCF10A cells were exposed to SC, CAPE, Torin-1, or LY294002, and 48 hours later, they were analyzed by immunoblot for either pAkt (S473), pAkt (T308), or β-tubulin. The data shown represents one of at least three experiments conducted. (D) HeLa and (E) A549 cells were infected with the indicated viruses, and four hpi they were exposed to AG1024, AG1478, SC, or CAPE at the indicated concentrations for 48 hours. The lysed cells were analyzed by immunoblot probing for IGF1R (Tot IGF1R) and phosphorylated IGF1R Tyr1135/1136 (pIGF1R). The normalized pIGF1R intensity values (S6 Table) were plotted in GraphPad Prism and are shown. The data shown represents one of at least three experiments conducted.

Inhibiting NF-κB transcriptional activity in Ad-infected cells disrupts their ability to accumulate abnormal DNA content [11]. In Figs 4, S2 and S4, we demonstrated that pharmacological agents such as AKTIV, AG1024, and, to a lesser extent, AG1478, which block Akt phosphorylation, also reduce the phosphorylation of NF-κB p50 at S337. The S337 phosphorylation of NF-κB p50 is necessary for its DNA binding activity [50]. This suggests that NF-κB acts downstream of Akt in cells infected with Ad. To further investigate the relationship between NF-κB and Akt, we exposed Ad-infected cells to the NF-κB inhibitors SC and CAPE separately (Fig 5A and 5B). The effects of AG1024 and AG1478 are presented in Figs 4 and S5. and are used here for comparison. As shown, SC was as effective as the IGF1R inhibitor at decreasing Akt phosphorylation in Ad-infected cells (Fig 5A and 5B). CAPE was less effective than SC, likely because of the differences in mode of action. SC blocks NF-κB DNA binding, while CAPE blocks its nuclear translocation. These results show that NF-κB activity is essential for Akt phosphorylation in Ad-infected cancer cells.

To extend our findings to “normal” cells, we treated MCF10A cells expressing E4orf1 with SC and CAPE, using LY294002 and Torin-1 for comparison. Consistent with results from A549 and HeLa cells (Figs 3, 4, 5A, 5B, S2 and S5), LY294002 did not reduce the phosphorylation of Akt in the *E4orf1*-expressing MCF10A cells (Fig 5C). Additionally, as shown in S4 Fig, Torin-1 reduced the phosphorylation of Akt S473 without changing the levels of phosphorylated T308 (Fig 5C). SC reduced the phosphorylation of Akt more effectively than CAPE (Fig 5C). Thus, NF-κB is essential for the phosphorylation of Akt in E4orf1-expressing “normal” cells as well.

We established in Fig 4 that IGF1R activation is crucial for Akt phosphorylation in Ad-infected cells. Therefore, we investigated the effect of NF-κB inhibition on IGF1R phosphorylation. In cells infected with *E4orf1(+)* Ads (both *E1B55K*-deleted on the left and wild-type Ad on the right), IGF1R levels were lower compared to those in the mock and cells infected with *E4orf1(-)* Ad (Fig 5D and 5E). However, after normalization and consistent with Fig 3, the plotted values for phosphorylated IGF1R were highest in *E4orf1(+)* cells compared to those infected with *E4orf1(-)* Ad (bottom of Fig 5D and 5E). Notably, here, too, SC reduced IGF1R phosphorylation similarly to AG1024, while CAPE produced results comparable to AG1478 (bottom of Fig 5D and 5E, *E1B55K*-deleted on the left and wild-type Ad on the right). Therefore, the pivotal role of NF-κB concerning abnormal DNA content [11] is likely due to its support for the phosphorylation of IGF1R and Akt in Ad-infected cells.

## Discussion

Receptor tyrosine kinases (RTKs), such as the EGFR and IGF1R, transmit signals from outside the cell that control cell cycle progression and metabolism. Due to gene amplification, overexpression, and mutation, their misfiring can lead to the development of cancer. Abnormal DNA content, such as aneuploidy and polyploidy, is recognized as a precursor to cancer, readily observed in infections with tumor viruses [6,7,9]. Ads are prototypical tumor viruses that have been shown to cause cancer [5]. We show here that RTKs can promote abnormal cellular DNA content [53,54]. In the Ad-infected cell, this is linked to E4orf1.

E4orf1, in the absence of other adenoviral genes, can stimulate the phosphorylation and activation of Akt. Activating Akt at levels similar to those induced by E4orf1 may be beneficial, as it creates a niche that allows primary cells to survive without serum or cytokines [55]. This ability of E4orf1 can be harnessed to extend the life of CAR T and CAR-NK cells (https://patentimages.storage.googleapis.com/48/71/9b/3a07635b9ad458/WO2023070079A1.pdf), which are currently being explored as tumor therapy [56,57]. E4orf1 can also improve metabolism [58–60]. Thus, it is being explored for diabetes treatment [61], for improving cognition in Alzheimer’s disease [62], and for reducing aging markers [63].

In light of our findings, we recast the roles of the Ad oncogenes E1A, E1B, E4orf6, E4orf3, and E4orf1 [64,65]. E1A’s interactions with pRB and p300 promote unscheduled DNA synthesis, leading to massive cell death [66–69]. Some cells capable of activating ancient polyploidization programs [70,71] can survive and become transformed [72,73]. *E1B* and *E4orf6* enhance the number of E1A-expressing cells that transform [64,65]. However, during infection, *E1B* and *E4orf6* prioritize viral progeny [20,21,74] over oncogenic transformation by increasing the availability of cytoplasmic viral RNA [74–77], thus minimizing the accumulation of cells with abnormal DNA content. Like E1B and E4orf6, E4orf3 also enables more E1A-transformed cells to survive [78]. E4orf3, by disrupting MRN complex formation, enhances levels of phosphorylated ATM [11]. Although our idea that ATM is active in Ad-infected cells differs from the mainstream view [78], the idea that ATM might be essential for fully activating Akt [79] and maintaining NF-κB levels [80–82] is supported by evidence showing that ATM is required for IGF1R expression [83,84] and for the phosphorylation and activation of Akt and NF-κB in Ad-infected cells [11]. This connection between E4orf3 and E4orf1 explains why E4orf1 activities increase in Ad-infected cells, but not when expressed alone.

**Fig 6:**
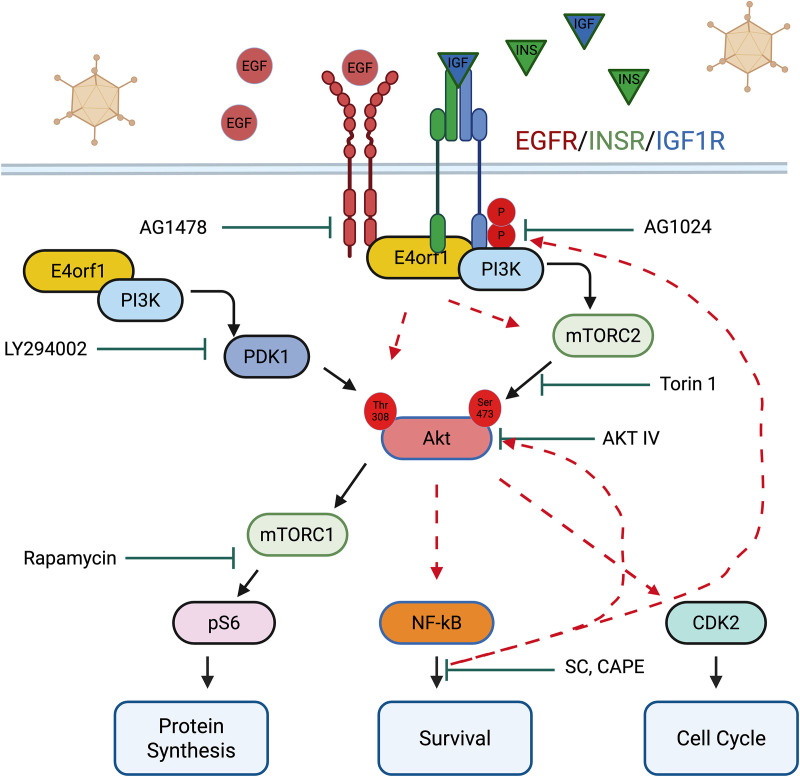
Schematic illustrating E4orf1-mediated signals that promote aneuploidy and polyploidy.

NF-κB has been reported to act downstream of Akt [79–81]. However, our study also suggests that NF-κB may function upstream of Akt (Fig 6). While this concept is new for Ad, the idea that NF-κB can act upstream of Akt may not be novel. In one study, overexpression of NF-κB resulted in increased Akt mRNA and protein expression [82]. In another, a positive feedback loop involving EGFR/Akt/mTORC1 and IKK/NF-κB was shown to influence proliferation in head and neck squamous cell carcinoma [83]. E4orf1, therefore, due to its capacity to integrate signals from various RTKs, may help maintain this Akt-NF-κB feedback loop, allowing polyploidization and transforming normal cells into cancer cells [15,27]. We used different drugs with distinct mechanisms of action for each pathway to minimize off-target or unintended effects; however, this remains a limitation. Nonetheless, while the signals we discussed may be necessary for E4orf1 to act as an oncogene, it is important to note that abnormal DNA content (aneuploidy and polyploidy) is also associated with aging and neurodegenerative diseases [84–87]. The signals described here (Fig 6) could also be relevant in those contexts.

In the presence of EGF, INS, and IGF1, and during Ad infection, E4orf1 activates a [PI3 kinase-dependent and independent] feedback loop involving EGFR, INSR, IGF1R, Akt, and NF-kB that enhances essential cellular activities, such as protein synthesis, cell survival, and progression through the cell cycle, all of which are vital for accumulating abnormal DNA content. (This Image was created in BioRender. M. A. Thomas. (2025) https://BioRender.com/ul94kp1

## Materials and methods

### Ethics statement

All experiments were approved by the Institutional Biosafety Committee (IBC) at Howard University (Approval # IBC-GSAS-19–08).

### Cells

We used the human cervical carcinoma-derived HeLa cell line (ATCC CCL-2) because most of what is known about Ad is in the context of this cell line. We used the human lung carcinoma epithelial A549 cell line, A549 (ATCC CCL-185), as Ad is known to target the upper respiratory airway. The HeLa and A549 cell lines were cultured in Dulbecco’s Modified Eagle *Medium* supplemented with 10%, 1%, or 0% Fetal Bovine Serum (FBS), 100 units/ml Penicillin, and 100 mg/ml Streptomycin. The human breast mammary epithelial cell line MCF10A, stably expressing either pBabe-empty or pBabe-E4orf1, was a gift from Ronald T. Javier, Baylor College of Medicine, Houston, Texas, and has been described previously [13,16]. MCF10A cells were cultured with DMEM/F12 medium supplemented with 5% horse serum (Life Technologies, Carlsbad, CA) and MEGM Mammary Epithelial Cell Growth Medium SingleQuots Kit (Lonza Cat # CC-4136). All cells were incubated at 37°C in humidified air with 5% CO₂.

### Viruses

The adenovirus subtype 5 (Ad5) host range mutant Mad5rhFLSC and the *E1B55K*- and *E4orf1*-deleted Ad have been described previously [24]. The Ad5 *dl*309 is another commonly used phenotypic wild-type Ad that has a deletion in the early region 3 (E3) [22,23]. Ad5 in351 contains a 5-base pair insertion within the *E4orf1* gene, which disallows the expression of the E4orf1 [74]. The *E1B55K*-deleted Ad5 mutant *dl*1520 has an 827-bp deletion in the region encoding the 55kDa protein [20]. The mutant Ad5 *dl3*55* contains a 14-bp deletion that inactivates E4orf6 [21]. The Ad *dl*1016 lacks E1B55K and E4orf1, E4orf2, and E4orf3 [30]. These viruses can be classified, as shown in Table 1, as either *E4orf1(+)* or *E4orf1(-)* Ad.

### Infection

Cells were plated in 6-well plates at a density of 5 × 10^^5^ cells per well or in 12-well plates at a density of 1.5 × 10^^5^ cells per well, and then infected at an MOI of 25 with the indicated Ads by incubating for 1 hour at 37°C, while rocking every 10–15 minutes. The media was aspirated and replaced with 2 mL (6-well dish) or 1 mL (12-well dish) of fresh medium and incubated for the specified time.

### Flow cytometry

Cells were detached from the plate using trypsin, washed in 1x PBS, and fixed in 70% ethanol overnight at -20$\backslash {(}^{\circ }\text{C}$. The fixed cells were then washed twice with PBS. After centrifuging, the pellet was stained with 500 μL of FxCycle PI/RNase staining solution (ThermoFisher Cat #: F10797) per 1 million cells. The samples were incubated at room temperature for 15–30 minutes, protected from light. The cells were acquired with a BD (Becton, Dickinson) FACSVerse flow cytometer. The population of cells in each phase of the cell cycle was quantified using FlowJo v10.8 Software (BD Life Sciences), as shown in S1A Fig.

### Western blot

Cells were lysed in 1X SDS sample buffer (ThermoFisher Cat #: LC2675–4) containing protease/phosphatase inhibitor (ThermoFisher Cat #: 78442) and 5% β-mercaptoethanol (BME). Equal amounts of lysate were loaded into wells of 4–20% Tris-Glycine gels (ThermoFisher Cat #: XP04205BOX). Following electrophoresis, proteins were transferred from the gel to a nitrocellulose membrane (ThermoFisher Cat #: IB23002). At room temperature, the membrane was blocked in PBS with 0.02% Tween-20 and 20% milk for one h. After blocking, the membrane was incubated in appropriate primary antibody dilutions in PBS with 0.02% Tween-20 and 10% milk on a rocker at four °C overnight. After washing three times in PBS with 0.02% Tween-20 and 5% milk for 5 min each time, the membrane was incubated with appropriate secondary antibody dilutions in PBS with 0.02% Tween-20 and 10% milk for 30 minutes at room temperature. The membrane was washed three times, for 5 minutes each, and then exposed to a 1:1 substrate luminal/enhancer solution. The image was captured using a LI-COR Odyssey Fc Imaging System (Lincoln, Nebraska, USA).

In each case, the blots were obtained by probing for one antigen, stripping with Restore Western Blot Stripping Buffer (ThermoFisher Cat #: 21059), and then probing again for another antigen after washing and blocking as described above. Where indicated, the Ad DNA binding protein (DBP) was used as an infection control, and Beta (β)-tubulin as a loading control. The lane denoting the molecular weight marker, MW, is on most blots.

### Western blot normalization

Fluorescent intensity was measured using the LI-Cor Image Studio Lite program. Each blotted area was manually gated, and intensity values were transferred to Excel. The lane normalization factor was determined by dividing each lane’s observed IGF1R signal intensity by the highest IGF1R fluorescent value on the membrane. The pIGF1R signals were divided by the lane normalization value. The normalized values were plotted using GraphPad Prism.

### Antibodies

The antibodies used for western blot and the dilutions used were Akt ser473 (Invitrogen Cat #: 44- 62G), 1:1000; Akt thr308 (Cell signaling Clone #: D25E6), 1:1000; Beta Tubulin (Invitrogen Cat #: PA1–16947), 1:5000; Cyclin A (BD Cat #: 611268), 1:1000; CDK2 T160 (Santa Cruz Biotech Cat #: SC-101656), 1:250; cyclin E (BD Cat:51–1459GR), 1:1000; IGF1R (Invitrogen Cat #: PA5–85986), 1:1000; HA Tag (Invitrogen Cat #: 26183), 1:5000; p50ser337 (Santa Cruz Biotech Cat #: SC-271908), 1:1000; p-S6 Ribosomal Protein S240/244 (Cell signaling Clone #: D68F8), 1:5000; P-p44/42 MapK T202/Y204 (cell signaling Clone #: D13.14.4E), 1:5000; cyclin B (BD Cat #: 610219), 1:1000; and P-IGF-1R beta Y1135/1136/INSR beta (Cell signaling Clone #: 19H7), 1:1000. The western blot marker used was the LI-COR molecular weight marker 928–40000.

### Pharmacological inhibitors

The pharmacological inhibitors used in this study were Torin1 (Cayman Chemical Company Cat #: 10997), LY294002 (Selleckchem Cat #: S1105), Akt inhibitor IV (Millipore Cat #: 124011), SC75741 (Selleckchem Cat #: S7273), AG-1478 (Selleckchem Cat #: S2728), AG-1024 (Selleckchem Cat #: S1234), Caffeic Acid Phenethyl Ester (CAPE) (Selleckchem Cat #: 7414), and rapamycin (Selleckchem Cat #: S1039).

### Plasmid transfection

The plasmids MSCV-N E4orf1 (Addgene plasmid # 38063) and MSCV-N GFP (Addgene plasmid # 37855) were gifts from Karl Munger and were described before [29]. Bacterial stabs were streaked on LB agar plates (Quality Biological Cat #: 340-1070-231) containing 50μg/ml of ampicillin, which were then incubated for 12 hours at 37°C. Following incubation, individual colonies were picked up using a sterile tip and placed in a 50 mL conical tube containing 25 mL of LB broth (KD Medical Cat #: BLF-7030), supplemented with 100 μg/mL of ampicillin. The tube was then placed in a shaker for 12 hours at 250 rpm at 37°C for bacterial amplification. Following shaker incubation, plasmid DNA was isolated using the Pure Link HiPure Plasmid Filter Maxiprep Kit (ThermoFisher Cat #: K210017), yielding pure plasmid DNA. The purified DNA was then transfected into cells using Lipofectamine 2000. After 24 hours, the transfected cells were incubated in 1 μM of puromycin for selection. Immunofluorescence microscopy was used to verify GFP expression, and a western blot was used to verify the expression of HA-tagged GFP and HA-tagged E4orf1.

### Immunofluorescence

Cells were seeded on coverslips at a density of 2.5 × 10^5 cells/well in 12-well plates and incubated at 37°C for 24 hours. Cells were washed twice with 1x PBS and fixed with 4% paraformaldehyde for 15 minutes at room temperature. Subsequently, cells were permeabilized in 0.5% TritonX-100 (PBS) for 10 minutes at room temperature. The cells were rinsed with 1x PBS and stained with DAPI Fluoromount-G (Invitrogen Cat #: 00-4959-52). Images were obtained using a Nikon Ti-E-PFS inverted microscope with a 100 × 1.4 NA Plan Apo Lambda objective. The microscope also had a Yokogawa CSU-X1 spinning disk unit and an Andor iXon 897 EMCCD camera. To analyze the DNA and GFP in both HeLa and A549 cells, excitations were selected at 405 nm and 488 nm, respectively. All images are overlay z-plates.

### Statistical analysis

Two-way analysis of variance (ANOVA) was used, along with Holm-Sidak’s multiple comparisons test, to compare mean differences in cell cycle phases between the groups. P-values < 0.05 are considered significant.

## Supporting information

S1 Fig Effects of Ad on the number of infected cells with abnormal DNA content. **(A)** HeLa cells were either mock or infected at a multiplicity of 30 plaque-forming units per cell (MOI 30 pfu/cell) with an *E1B55K*-deleted Ad (*∆E1B*) or an *E1B55K*- and E4orf1-deleted Ad (*∆E1B/E4orf1*). At 48 hours post-infection (hpi), the cells were washed, stained with an RNase-containing propidium iodide solution, and analyzed by flow cytometry. Propidium iodide (PI) binds to cellular DNA, enabling the determination of different cell cycle phases [20,21]. (**i**) Cells gated to exclude doublets and other clumps that could interfere with the results. (**ii, iii**) Most of the cultured cells (67.8%) are in the G1 phase. (**iii**) The percentage of cells with abnormal DNA content (DNA > 4n) is shown and highlighted in blue in this and all subsequent images. (**iv-v**) By 48 hpi, 61% of the *∆E1B* adenovirus-infected cells exhibited abnormal DNA content, compared to 32% of the *∆E1B/E4orf1*-infected cells. (**vi**) An overlay of the histograms from **ii-v** is shown. **(B)** Deletion of E4orf1 from wild-type Ad has little effect on DNA content. HeLa cells were infected at an MOI of 50 pfu/cell with **either (top)** wild-type Ad *dl*309 or **(bottom) Ad** *in*351, which has an insertion mutation in the *E4orf1* gene, for 12 and 24 hours. The percentage of cells with abnormal DNA content for each virus infection is shown in blue. **(C)** More cells infected with *E1B55K*- and *E4orf6*-deleted Ads have abnormal DNA content compared to cells infected with wild-type Ad. HeLa cells were infected at an MOI of 50 pfu/cell with **(top)** the *E1B55K*-deleted Ad*, dl*1520, or **(bottom)** an *E4orf6*-deleted Ad (*∆Eorf6*) *dl*355* for 12 and 24 hours. The stained cells were processed and analyzed as described in part A.
(TIFF)

S2 Fig PI3K and AKT signals are needed for cell cycle transition and the accumulation of abnormal DNA content in Ad-infected cells. (A) A549 cells were incubated in 1% FBS for 24 hours and then stimulated with 1μg/mL of insulin growth factor 1 (IGF1) for 30 minutes with or without the 50 μM PI3K inhibitor LY294002. The vehicle control (v cont) DMSO was used as a negative control. The lysed cells were analyzed by immunoblotting for pAkt (S473), pS6 (S235/S236), and β-tubulin. (B, D) A549 and (C, E) HeLa cells were infected at an MOI of 25pfu/cell with the indicated Ads and 4 hours post-infection (hpi) exposed to either 50 μM LY294002 or 5 μM AKTIV and allowed to incubate in 10% FBS for a total of 48 hours. (B, C) The lysed cells were analyzed for one or more of the following targets using immunoblotting: phosphorylated AKT (pAkt) S473, pAkt (T308), NF-κB p50 (S337), pS6 (S235/S236), pCdk2 (T160), pERK1/2 (T202/Y204), as well as cyclins A, B, and E, DBP, and β-tubulin. (D) For each group of A549 (n = 3–8) and (E) HeLa cells (n = 3–6), averages of the percent cells in each phase of the cell cycle (S3 Table) with their individual SEM were plotted in GraphPad Prism and are shown. The P values were calculated using a two-way analysis of variance (ANOVA) with Holm–Šídák’s multiple comparisons tests. (F, G) A549 cells were infected with the indicated viruses, and four hpi were exposed to either LY294002 or AKTIV at the indicated concentrations and allowed to incubate in 10% or 1% FBS for 48 hours. (F) The lysed cells were analyzed using immunoblotting, probing for pAkt (S473), pAkt (T308), NF-κB p50 (S337), pCdk2 (T160), cyclin A, cyclin B, cyclin E, DBP, and β-tubulin. (G) For each group (n = 3–8), averages of the percent cells in each phase of the cell cycle (S3 Table) with their individual SEM were plotted in GraphPad Prism and are shown. The P values were calculated using a two-way analysis of variance (ANOVA) with Holm–Šídák’s multiple comparisons test.
(TIF)

S3 Fig Summary statistics comparing the effects of different FBS concentrations on the cell cycle in Ad-infected A549 (top) and HeLa cells (bottom). The P values were calculated using a two-way analysis of variance (ANOVA) with Holm–Šídák’s multiple comparisons test.
(PDF)

S4 Fig The mTOR complexes 1 and 2 mediate abnormal DNA content in Ad-infected cells. (A) A549 cells were incubated in 1% FBS for 24 hours, followed by stimulation with 1 μg/mL of insulin growth factor 1 (IGF1) for 30 minutes, with or without 50 nM rapamycin or 500 ng/mL Torin1. DMSO served as a negative control. The lysed cells were analyzed by immunoblotting for pAkt (S473), pS6 (S235/S236), and β-tubulin. (B, C) A549 cells were infected with the specified viruses. Four hours post-infection (hpi), they were treated with either 50 nM rapamycin or 500 ng/mL Torin1 and allowed to incubate in (B) 10% or (C) 1% FBS for a total of 48 hours. The stained cells were analyzed by flow cytometry. For each group incubated in 10% (n = 4–6) and 1% FBS (n = 3–8), the average percentages of cells in each phase of the cell cycle, along with their respective standard error of the mean (SEM), were plotted using GraphPad Prism and are presented here. The P values were calculated using a two-way analysis of variance (ANOVA) with Holm–Šídák’s multiple comparisons test. (D, E) A549 cells were infected with the specified viruses, and four hours post-infection (hpi), they were exposed to either rapamycin or Torin1 at the indicated concentrations, followed by incubation in (D) 10% or (E) 1% FBS for 48 hours. The lysed cells were analyzed using immunoblotting to detect pAkt (S473), pAkt (T308), NF-κB p50 (S337), pCdk2 (T160), pS6 (S235/S236), cyclin A, cyclin B, cyclin E, DBP, and β-tubulin.
(TIF)

S5 Fig Effect of insulin and growth factor receptors on abnormal DNA content in Ad-infected cells. (A) A549 cells were incubated in 10% FBS for 24 hours and then stimulated with 500ng/mL of epithelial growth factor (EGF) for 30 minutes with or without 20uM of the epithelial growth factor receptor inhibitor AG1478. The lysed cells were analyzed by immunoblotting for pAkt (S473), pERK1/2 (T202/Y204), pS6 (S235/S236), and β-tubulin. (B) HeLa cells were infected with the below-indicated Ads, and four hpi were exposed to the indicated concentrations of AG1478 or AG1024 and incubated in 10% FBS for 48 hours. The stained cells were interrogated by flow cytometry. The averages (n = 5–9) of the percent cells in each phase of the cell cycle, with their individual SEM, were plotted in GraphPad Prism and are shown. The P values were calculated using a two-way analysis of variance (ANOVA) with Holm–Šídák’s multiple comparisons tests. (C, D) HeLa cells were infected with the indicated Ads. Four hpi, the cells were treated with specific inhibitors: the epithelial growth factor receptor inhibitor AG1478, the insulin-like growth factor 1 receptor inhibitor AG1024, the AKT inhibitor AKTIV, or the PI3K inhibitor LY294002 at designated concentrations. The cells were then incubated for a total of 48 hours in media containing either 10% or 1% FBS, as shown in (C) and (D), respectively. Afterwards, the lysed cells were subjected to immunoblotting analysis for pAkt (S473), NF-κB p50 (S337), pS6 (S235/S236), cyclin A, cyclin B, cyclin E, DBP, and β-tubulin. (E) A549 cells expressing HA-tagged GFP or HA-tagged E4orf1 were incubated in 0% FBS and infected with or without the *E4orf1(-)* Ad at an MOI of 25 pfu/cell. Four hpi, the cells were stimulated with 1 μg/mL IGF1, 20 μg/mL INS, or 500 ng/mL EGF. Twenty-four hours later, the stained cells were analyzed by flow cytometry. For each group (n = 2), the averages of the percentage of cells in each cell cycle phase, along with their standard deviations (SD), were plotted in GraphPad Prism and are presented.
(TIF)

S6 Fig Summary statistics comparing the effects of AG1024 and AG1478 on the cell cycle in mock and Ad-infected A549 cells in 10% FBS (top) and in 1% FBS (bottom). The P values were calculated using a two-way analysis of variance (ANOVA) with Holm–Šídák’s multiple comparisons test.
(PDF)

S1 Table Values for graphs in Fig 1. Percent GFP and E4orf1-expressing A549 (Fig 1B) and HeLa cells infected with Ad (Fig 1D) in each phase of the cell cycle.
(XLSX)

S2 Table Values for graphs in Fig 2. Percentages of A549 (Fig 2A) and HeLa Ad-infected cells (Fig 2D) in each cell cycle phase. These values were derived from experiments performed at specific MOI and time points throughout the study.
(XLSX)

S3 Table Values for graphs in S2 Fig. Percentages of A549 cells (grown in 10% FBS, S2D Fig, and 1% FBS, S2G Fig) and HeLa Ad-infected cells (grown in 10% FBS, S2E Fig) in each cell cycle phase. These values were obtained from experiments conducted at the specified MOI and time points throughout the study.
(XLSX)

S4 Table Values for graphs in Fig 3B. Arbitrary units obtained from normalized pIGF1R intensity.
(XLSX)

S5 Table Values for graphs in Fig 4. Percentages of A549 Ad-infected cells in each cell cycle phase (Fig 4A, grown in 10% FBS, and Fig 4B, 1% FBS). These values were obtained from experiments conducted at the specified MOI and time points throughout the study. This Excel sheet also includes the percentage of insulin- and growth factor-treated E4orf1(-) Ad-infected GFP-expressing and E4orf1-expressing HeLa cells (Fig 4F) in each cell cycle phase.
(XLSX)

S6 Table Values for graphs in Fig 5. Arbitrary units obtained from normalized pIGF1R intensity in HeLa cells (cultured with 10% FBS, Fig 5D) and A549 Ad-infected cells (cultured with 10% FBS, Fig 5E).
(XLSX)
