# Supplementary figures and images for: The adenovirus E4orf1 protein initiates a feedback loop involving insulin and growth factor receptors, AKT, and NF-κB, leading to abnormal DNA content in infected cells
Source: PLoS Pathog. 2025 Oct 27;21(10):e1013202. doi: 10.1371/journal.ppat.1013202 (PMC12578350; doi:10.1371/journal.ppat.1013202)

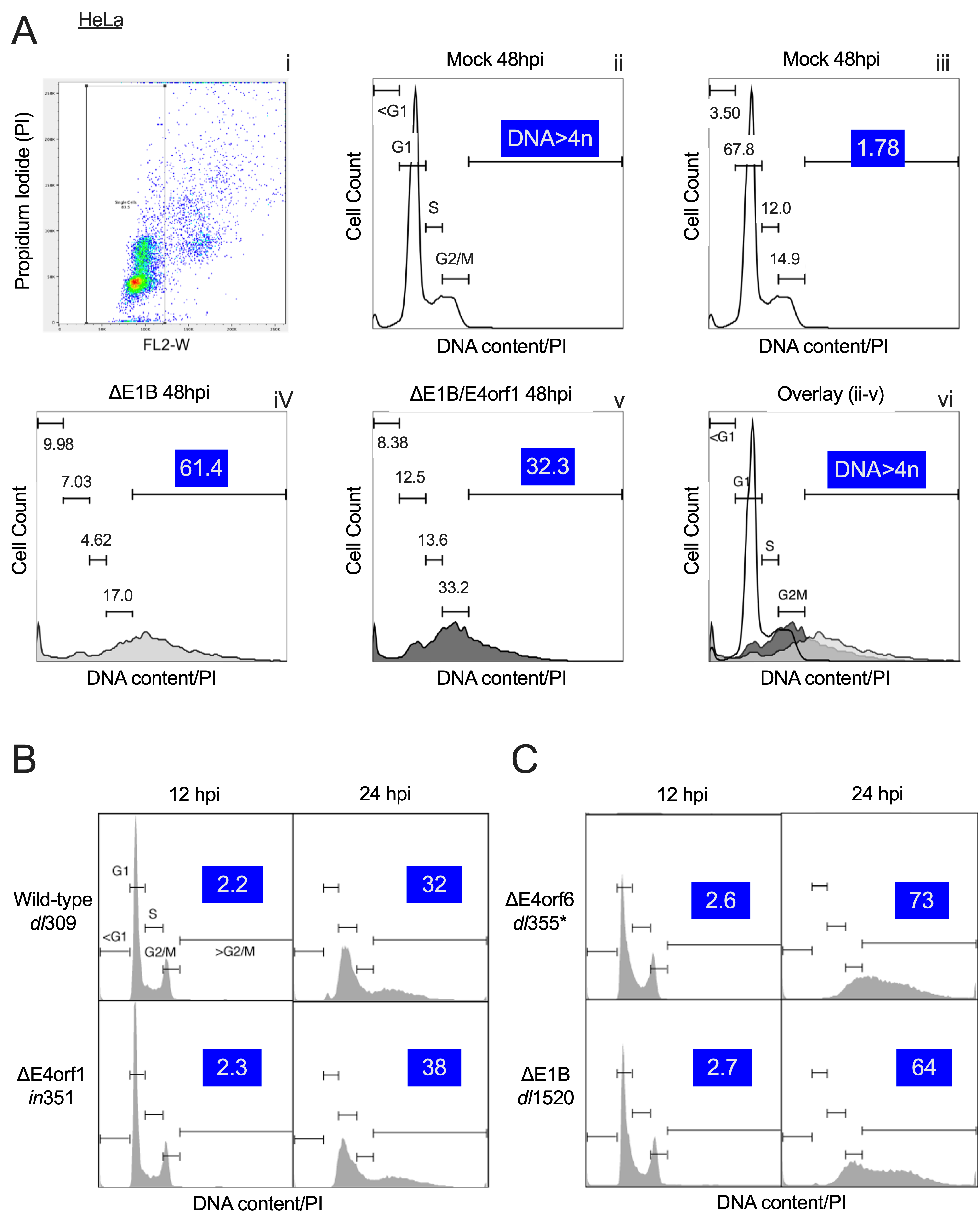

Supplement: S1 Fig — (A) HeLa cells were either mock or infected at a multiplicity of 30 plaque-forming units per cell (MOI 30 pfu/cell) with an E1B55K-deleted Ad (∆E1B) or an E1B55K- and E4orf1-deleted Ad (∆E1B/E4orf1). At 48 hours post-infection (hpi), the cells were washed, stained with an RNase-containing propidium iodide solution, and analyzed by flow cytometry. Propidium iodide (PI) binds to cellular DNA, enabling the determination of different cell cycle phases [20,21]. (i) Cells gated to exclude doublets and other clumps that could interfere with the results. (ii, iii) Most of the cultured cells (67.8%) are in the G1 phase. (iii) The percentage of cells with abnormal DNA content (DNA > 4n) is shown and highlighted in blue in this and all subsequent images. (iv-v) By 48 hpi, 61% of the ∆E1B adenovirus-infected cells exhibited abnormal DNA content, compared to 32% of the ∆E1B/E4orf1-infected cells. (vi) An overlay of the histograms from ii-v is shown. (B) Deletion of E4orf1 from wild-type Ad has little effect on DNA content. HeLa cells were infected at an MOI of 50 pfu/cell with either (top) wild-type Ad dl309 or (bottom) Ad in351, which has an insertion mutation in the E4orf1 gene, for 12 and 24 hours. The percentage of cells with abnormal DNA content for each virus infection is shown in blue. (C) More cells infected with E1B55K- and E4orf6-deleted Ads have abnormal DNA content compared to cells infected with wild-type Ad. HeLa cells were infected at an MOI of 50 pfu/cell with (top) the E1B55K-deleted Ad, dl1520, or (bottom) an E4orf6-deleted Ad (∆Eorf6) dl355* for 12 and 24 hours. The stained cells were processed and analyzed as described in part A. (TIFF) [file ppat.1013202.s001.tiff]

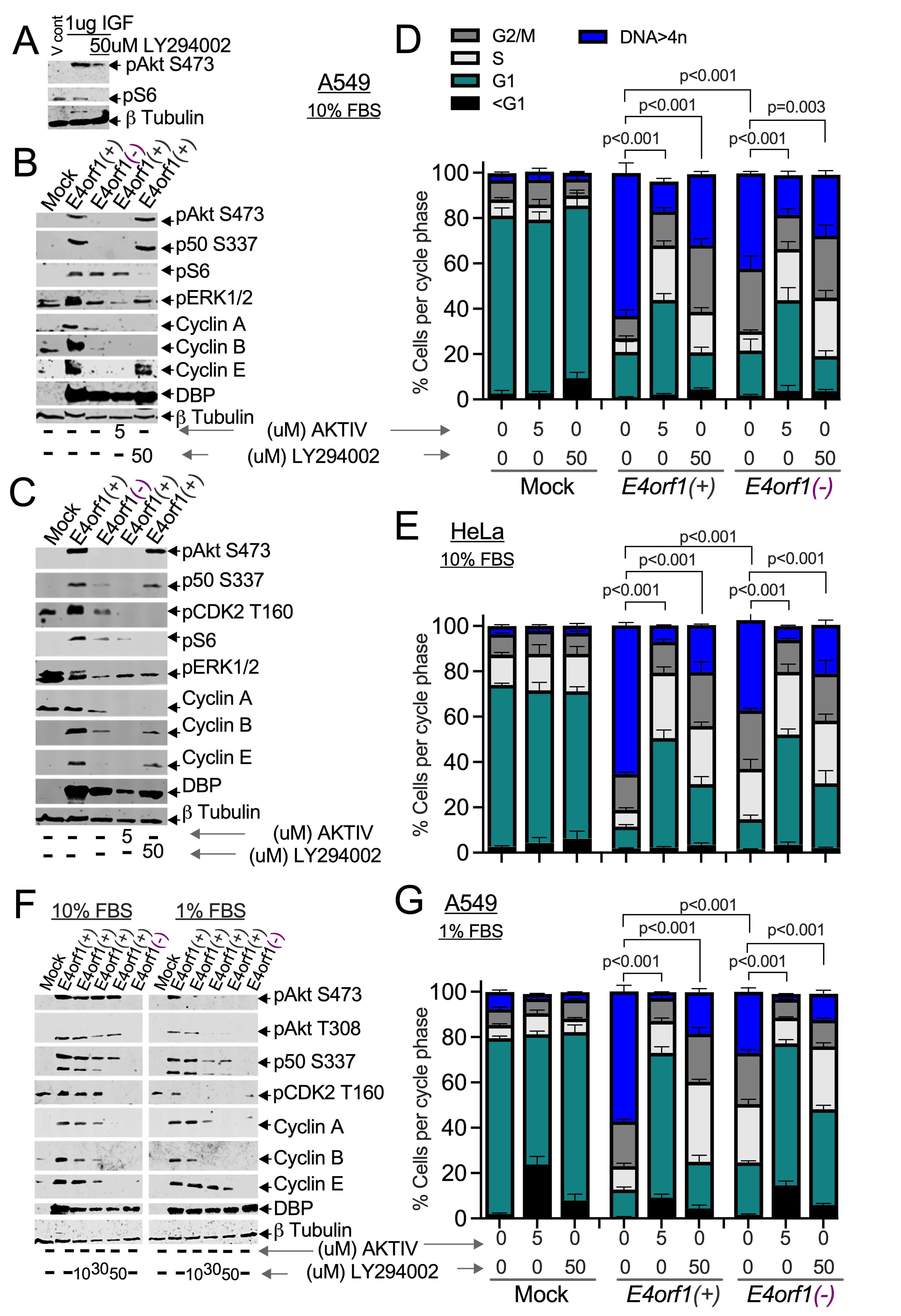

Supplement: S2 Fig — (A) A549 cells were incubated in 1% FBS for 24 hours and then stimulated with 1μg/mL of insulin growth factor 1 (IGF1) for 30 minutes with or without the 50 μM PI3K inhibitor LY294002. The vehicle control (v cont) DMSO was used as a negative control. The lysed cells were analyzed by immunoblotting for pAkt (S473), pS6 (S235/S236), and β-tubulin. (B, D) A549 and (C, E) HeLa cells were infected at an MOI of 25pfu/cell with the indicated Ads and 4 hours post-infection (hpi) exposed to either 50 μM LY294002 or 5 μM AKTIV and allowed to incubate in 10% FBS for a total of 48 hours. (B, C) The lysed cells were analyzed for one or more of the following targets using immunoblotting: phosphorylated AKT (pAkt) S473, pAkt (T308), NF-κB p50 (S337), pS6 (S235/S236), pCdk2 (T160), pERK1/2 (T202/Y204), as well as cyclins A, B, and E, DBP, and β-tubulin. (D) For each group of A549 (n = 3–8) and (E) HeLa cells (n = 3–6), averages of the percent cells in each phase of the cell cycle (S3 Table) with their individual SEM were plotted in GraphPad Prism and are shown. The P values were calculated using a two-way analysis of variance (ANOVA) with Holm–Šídák’s multiple comparisons tests. (F, G) A549 cells were infected with the indicated viruses, and four hpi were exposed to either LY294002 or AKTIV at the indicated concentrations and allowed to incubate in 10% or 1% FBS for 48 hours. (F) The lysed cells were analyzed using immunoblotting, probing for pAkt (S473), pAkt (T308), NF-κB p50 (S337), pCdk2 (T160), cyclin A, cyclin B, cyclin E, DBP, and β-tubulin. (G) For each group (n = 3–8), averages of the percent cells in each phase of the cell cycle (S3 Table) with their individual SEM were plotted in GraphPad Prism and are shown. The P values were calculated using a two-way analysis of variance (ANOVA) with Holm–Šídák’s multiple comparisons test. (TIF) [file ppat.1013202.s002.tif]

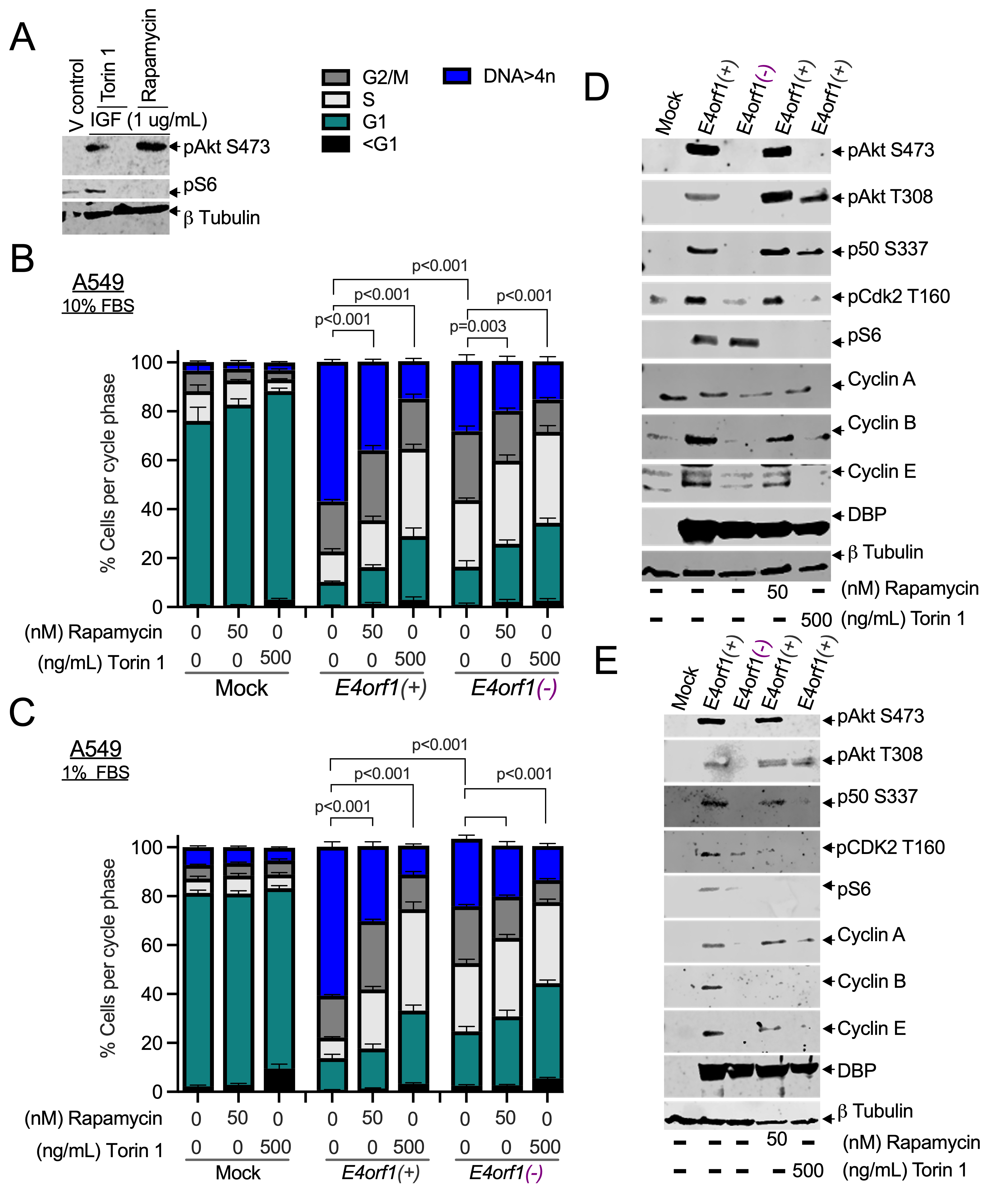

Supplement: S4 Fig — (A) A549 cells were incubated in 1% FBS for 24 hours, followed by stimulation with 1 μg/mL of insulin growth factor 1 (IGF1) for 30 minutes, with or without 50 nM rapamycin or 500 ng/mL Torin1. DMSO served as a negative control. The lysed cells were analyzed by immunoblotting for pAkt (S473), pS6 (S235/S236), and β-tubulin. (B, C) A549 cells were infected with the specified viruses. Four hours post-infection (hpi), they were treated with either 50 nM rapamycin or 500 ng/mL Torin1 and allowed to incubate in (B) 10% or (C) 1% FBS for a total of 48 hours. The stained cells were analyzed by flow cytometry. For each group incubated in 10% (n = 4–6) and 1% FBS (n = 3–8), the average percentages of cells in each phase of the cell cycle, along with their respective standard error of the mean (SEM), were plotted using GraphPad Prism and are presented here. The P values were calculated using a two-way analysis of variance (ANOVA) with Holm–Šídák’s multiple comparisons test. (D, E) A549 cells were infected with the specified viruses, and four hours post-infection (hpi), they were exposed to either rapamycin or Torin1 at the indicated concentrations, followed by incubation in (D) 10% or (E) 1% FBS for 48 hours. The lysed cells were analyzed using immunoblotting to detect pAkt (S473), pAkt (T308), NF-κB p50 (S337), pCdk2 (T160), pS6 (S235/S236), cyclin A, cyclin B, cyclin E, DBP, and β-tubulin. (TIF) [file ppat.1013202.s004.tif]

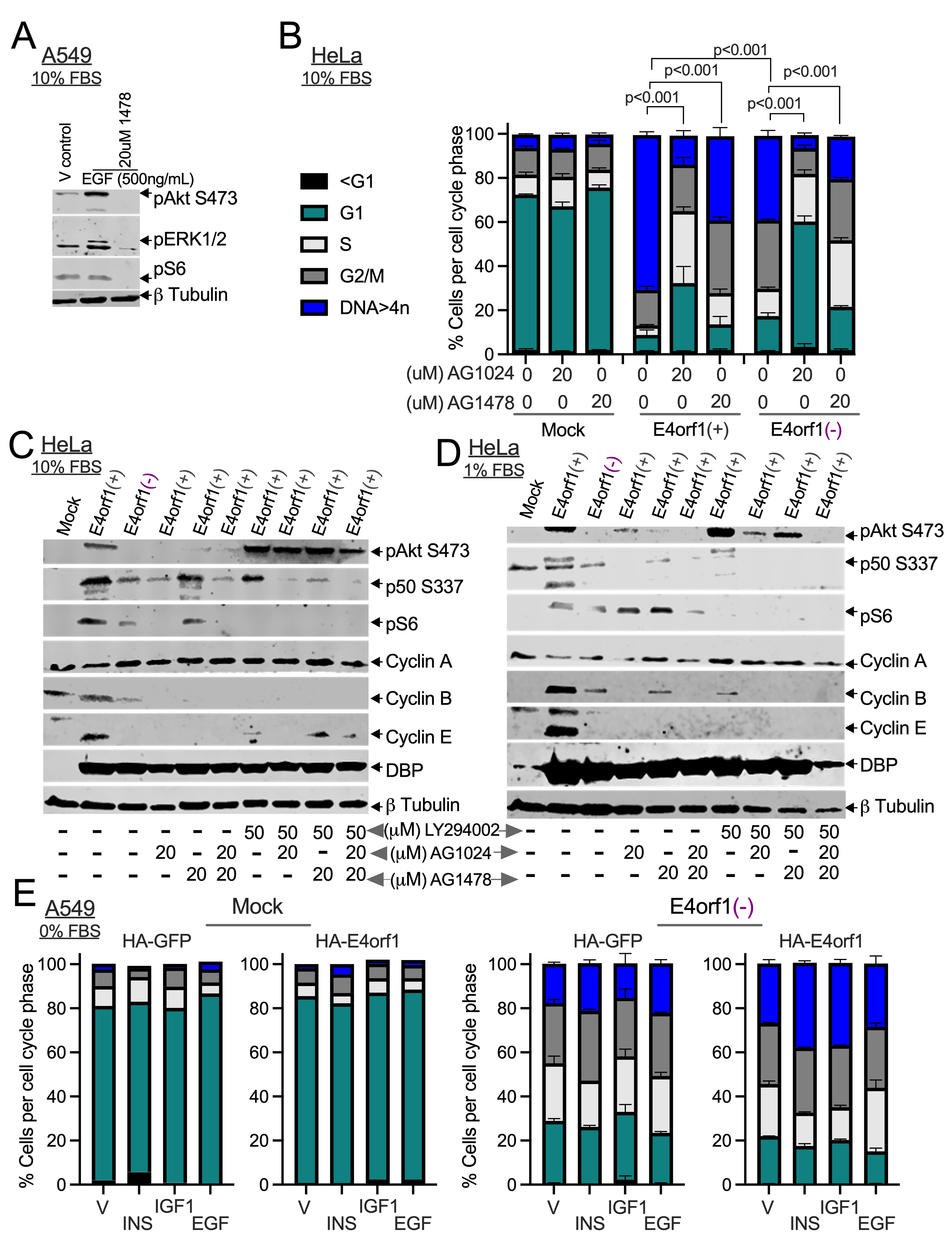

Supplement: S5 Fig — (A) A549 cells were incubated in 10% FBS for 24 hours and then stimulated with 500ng/mL of epithelial growth factor (EGF) for 30 minutes with or without 20uM of the epithelial growth factor receptor inhibitor AG1478. The lysed cells were analyzed by immunoblotting for pAkt (S473), pERK1/2 (T202/Y204), pS6 (S235/S236), and β-tubulin. (B) HeLa cells were infected with the below-indicated Ads, and four hpi were exposed to the indicated concentrations of AG1478 or AG1024 and incubated in 10% FBS for 48 hours. The stained cells were interrogated by flow cytometry. The averages (n = 5–9) of the percent cells in each phase of the cell cycle, with their individual SEM, were plotted in GraphPad Prism and are shown. The P values were calculated using a two-way analysis of variance (ANOVA) with Holm–Šídák’s multiple comparisons tests. (C, D) HeLa cells were infected with the indicated Ads. Four hpi, the cells were treated with specific inhibitors: the epithelial growth factor receptor inhibitor AG1478, the insulin-like growth factor 1 receptor inhibitor AG1024, the AKT inhibitor AKTIV, or the PI3K inhibitor LY294002 at designated concentrations. The cells were then incubated for a total of 48 hours in media containing either 10% or 1% FBS, as shown in (C) and (D), respectively. Afterwards, the lysed cells were subjected to immunoblotting analysis for pAkt (S473), NF-κB p50 (S337), pS6 (S235/S236), cyclin A, cyclin B, cyclin E, DBP, and β-tubulin. (E) A549 cells expressing HA-tagged GFP or HA-tagged E4orf1 were incubated in 0% FBS and infected with or without the E4orf1(-) Ad at an MOI of 25 pfu/cell. Four hpi, the cells were stimulated with 1 μg/mL IGF1, 20 μg/mL INS, or 500 ng/mL EGF. Twenty-four hours later, the stained cells were analyzed by flow cytometry. For each group (n = 2), the averages of the percentage of cells in each cell cycle phase, along with their standard deviations (SD), were plotted in GraphPad Prism and are presented. (TIF) [file ppat.1013202.s005.tif]
