# Supplementary material for: The adenovirus E4orf1 protein initiates a feedback loop involving insulin and growth factor receptors, AKT, and NF-κB, leading to abnormal DNA content in infected cells
Source: PLoS Pathog. 2025 Oct 27;21(10):e1013202. doi: 10.1371/journal.ppat.1013202 (PMC12578350; doi:10.1371/journal.ppat.1013202)
Supplement: S3 Fig — The P values were calculated using a two-way analysis of variance (ANOVA) with Holm–Šídák’s multiple comparisons test. (PDF) [file ppat.1013202.s003.pdf]

Summary stats for Fig 2A comparing the effects of various FBS concentrations on the cell cycle in mock and Ad-infected A549 cells.

Holm-Šídák's multiple comparisons test	<G1		G1		S		G2/M		DNA>4n	
n=4-11	Summary	Adjusted P	Summary	Adjusted P	Summary	Adjusted P	Summary	Adjusted P	Summary	Adjusted P
Mock 10% vs. Mock 1%	ns	>0.99	ns	0.95	ns	0.97	ns	>0.99	ns	0.97
Mock 10% vs. Mock 0%	ns	>0.99	ns	0.98	ns	>0.99	ns	>0.99	ns	0.97
Mock 10% vs. ΔE1B 10%	ns	>0.99	***	<0.001	ns	>0.99	ns	>0.99	***	<0.001
Mock 10% vs. ΔE1B 1%	ns	>0.99	***	<0.001	ns	0.97	ns	0.16	***	<0.001
Mock 10% vs. ΔE1B 0%	ns	>0.99	***	<0.001	ns	0.28	***	<0.001	***	<0.001
Mock 10% vs. ΔE4orf1 10%	ns	>0.99	***	<0.001	ns	0.89	***	<0.001	***	<0.001
Mock 10% vs. ΔE4orf1 1%	ns	>0.99	***	<0.001	ns	0.15	***	<0.001	***	<0.001
Mock 10% vs. ΔE4orf1 0%	ns	>0.99	***	<0.001	***	<0.001	***	<0.001	***	<0.001
Mock 1% vs. Mock 0%	ns	>0.99	ns	0.93	ns	>0.99	ns	>0.99	ns	0.99
Mock 1% vs. ΔE1B 10%	ns	>0.99	***	<0.001	ns	>0.99	ns	0.95	***	<0.001
Mock 1% vs. ΔE1B 1%	ns	>0.99	***	<0.001	ns	>0.99	*	0.03	***	<0.001
Mock 1% vs. ΔE1B 0%	ns	>0.99	***	<0.001	*	0.01	***	<0.001	***	<0.001
Mock 1% vs. ΔE4orf1 10%	ns	>0.99	***	<0.001	ns	0.16	***	<0.001	***	<0.001
Mock 1% vs. ΔE4orf1 1%	ns	>0.99	***	<0.001	**	0.004	***	<0.001	***	<0.001
Mock 1% vs. ΔE4orf1 0%	ns	>0.99	***	<0.001	***	<0.001	***	<0.001	***	<0.001
Mock 0% vs. ΔE1B 10%	ns	>0.99	***	<0.001	ns	>0.99	ns	>0.99	***	<0.001
Mock 0% vs. ΔE1B 1%	ns	>0.99	***	<0.001	ns	>0.99	ns	0.45	***	<0.001
Mock 0% vs. ΔE1B 0%	ns	>0.99	***	<0.001	ns	0.15	***	<0.001	***	<0.001
Mock 0% vs. ΔE4orf1 10%	ns	>0.99	***	<0.001	ns	0.66	***	<0.001	***	<0.001
Mock 0% vs. ΔE4orf1 1%	ns	>0.99	***	<0.001	ns	0.07	***	<0.001	***	<0.001
Mock 0% vs. ΔE4orf1 0%	ns	>0.99	***	<0.001	***	<0.001	***	<0.001	***	<0.001
ΔE1B 10% vs. ΔE1B 1%	ns	>0.99	ns	0.2	ns	>0.99	ns	0.25	ns	0.72
ΔE1B 10% vs. ΔE1B 0%	ns	>0.99	*	0.02	**	0.009	***	<0.001	***	<0.001
ΔE1B 10% vs. ΔE4orf1 10%	ns	>0.99	ns	0.95	ns	0.15	***	<0.001	***	<0.001
ΔE1B 10% vs. ΔE4orf1 1%	ns	>0.99	ns	0.9	**	0.002	***	<0.001	***	<0.001
ΔE1B 10% vs. ΔE4orf1 0%	ns	>0.99	ns	0.98	***	<0.001	***	<0.001	***	<0.001
ΔE1B 1% vs. ΔE1B 0%	ns	>0.99	ns	0.95	**	0.002	**	0.008	***	<0.001
ΔE1B 1% vs. ΔE4orf1 10%	ns	>0.99	*	0.02	ns	0.06	***	<0.001	***	<0.001
ΔE1B 1% vs. ΔE4orf1 1%	ns	>0.99	**	0.005	***	<0.001	***	<0.001	***	<0.001
ΔE1B 1% vs. ΔE4orf1 0%	ns	>0.99	ns	0.23	***	<0.001	***	<0.001	***	<0.001
ΔE1B 0% vs. ΔE4orf1 10%	ns	>0.99	**	0.001	ns	0.97	ns	>0.99	*	0.02
ΔE1B 0% vs. ΔE4orf1 1%	ns	>0.99	***	<0.001	ns	>0.99	ns	0.55	***	<0.001
ΔE1B 0% vs. ΔE4orf1 0%	ns	>0.99	*	0.03	**	0.002	ns	>0.99	***	<0.001
ΔE4orf1 10% vs. ΔE4orf1 1%	ns	>0.99	ns	0.98	ns	0.89	ns	0.95	*	0.02
ΔE4orf1 10% vs. ΔE4orf1 0%	ns	>0.99	ns	0.95	***	<0.001	ns	>0.99	***	<0.001
ΔE4orf1 1% vs. ΔE4orf1 0%	ns	>0.99	ns	0.93	**	0.001	ns	0.96	*	0.02

Summary stats for Fig 2D comparing the effects of various FBS concentrations on the cell cycle in mock and Ad-infected HeLa cells.

Holm-Šídák's multiple comparisons test	<G1		G1		S		G2/M		DNA>4n	
n=4-6	Summary	Adjusted P	Summary	Adjusted P	Summary	Adjusted P	Summary	Adjusted P	Summary	Adjusted P
Mock 10% vs. Mock 1%	ns	>0.99	ns	0.93	ns	0.98	ns	0.99	ns	0.91
Mock 10% vs. Mock 0%	ns	>0.99	ns	0.93	ns	0.98	ns	0.95	ns	0.91
Mock 10% vs. ΔE1B 10%	ns	>0.99	***	<0.001	ns	0.98	***	<0.001	***	<0.001
Mock 10% vs. ΔE1B 1%	ns	>0.99	***	<0.001	ns	0.86	***	<0.001	***	<0.001
Mock 10% vs. ΔE1B 0%	ns	>0.99	***	<0.001	ns	0.78	***	<0.001	***	<0.001
Mock 10% vs. ΔE4orf1 10%	ns	>0.99	***	<0.001	**	0.001	***	<0.001	***	<0.001
Mock 10% vs. ΔE4orf1 1%	ns	>0.99	***	<0.001	***	<0.001	***	<0.001	***	<0.001
Mock 10% vs. ΔE4orf1 0%	ns	>0.99	***	<0.001	***	<0.001	***	<0.001	***	<0.001
Mock 1% vs. Mock 0%	ns	>0.99	ns	0.93	ns	>0.99	ns	0.99	ns	0.93
Mock 1% vs. ΔE1B 10%	ns	>0.99	***	<0.001	ns	0.92	***	<0.001	***	<0.001
Mock 1% vs. ΔE1B 1%	ns	>0.99	***	<0.001	ns	0.98	***	<0.001	***	<0.001
Mock 1% vs. ΔE1B 0%	ns	>0.99	***	<0.001	ns	0.98	***	<0.001	***	<0.001
Mock 1% vs. ΔE4orf1 10%	ns	>0.99	***	<0.001	*	0.02	***	<0.001	***	<0.001
Mock 1% vs. ΔE4orf1 1%	ns	>0.99	***	<0.001	***	<0.001	***	<0.001	***	<0.001
Mock 1% vs. ΔE4orf1 0%	ns	>0.99	***	<0.001	***	<0.001	***	<0.001	***	<0.001
Mock 0% vs. ΔE1B 10%	ns	>0.99	***	<0.001	ns	0.93	**	0.005	***	<0.001
Mock 0% vs. ΔE1B 1%	ns	>0.99	***	<0.001	ns	0.98	***	<0.001	***	<0.001
Mock 0% vs. ΔE1B 0%	ns	>0.99	***	<0.001	ns	0.98	***	<0.001	***	<0.001
Mock 0% vs. ΔE4orf1 10%	ns	>0.99	***	<0.001	*	0.03	***	<0.001	***	<0.001
Mock 0% vs. ΔE4orf1 1%	ns	>0.99	***	<0.001	***	<0.001	***	<0.001	***	<0.001
Mock 0% vs. ΔE4orf1 0%	ns	>0.99	***	<0.001	***	<0.001	***	<0.001	***	<0.001
ΔE1B 10% vs. ΔE1B 1%	ns	>0.99	ns	0.17	ns	0.39	***	<0.001	***	<0.001
ΔE1B 10% vs. ΔE1B 0%	ns	>0.99	ns	0.39	ns	0.28	***	<0.001	***	<0.001
ΔE1B 10% vs. ΔE4orf1 10%	ns	>0.99	ns	0.93	***	<0.001	***	<0.001	***	<0.001
ΔE1B 10% vs. ΔE4orf1 1%	ns	>0.99	*	0.03	***	<0.001	ns	0.28	***	<0.001
ΔE1B 10% vs. ΔE4orf1 0%	ns	>0.99	***	<0.001	***	<0.001	ns	0.28	***	<0.001
ΔE1B 1% vs. ΔE1B 0%	ns	>0.99	ns	0.93	ns	0.98	ns	>0.99	ns	0.91
ΔE1B 1% vs. ΔE4orf1 10%	ns	>0.99	*	0.03	ns	0.11	**	0.007	ns	0.07
ΔE1B 1% vs. ΔE4orf1 1%	ns	>0.99	***	<0.001	***	<0.001	***	<0.001	***	<0.001
ΔE1B 1% vs. ΔE4orf1 0%	ns	>0.99	***	<0.001	***	<0.001	***	<0.001	***	<0.001
ΔE1B 0% vs. ΔE4orf1 10%	ns	>0.99	ns	0.12	ns	0.16	**	0.006	ns	0.42
ΔE1B 0% vs. ΔE4orf1 1%	ns	>0.99	***	<0.001	***	<0.001	***	<0.001	***	<0.001
ΔE1B 0% vs. ΔE4orf1 0%	ns	>0.99	***	<0.001	***	<0.001	***	<0.001	***	<0.001
ΔE4orf1 10% vs. ΔE4orf1 1%	ns	>0.99	ns	0.17	***	<0.001	ns	0.23	***	<0.001
ΔE4orf1 10% vs. ΔE4orf1 0%	ns	>0.99	***	<0.001	***	<0.001	ns	0.25	***	<0.001
ΔE4orf1 1% vs. ΔE4orf1 0%	ns	>0.99	*	0.02	ns	0.78	ns	>0.99	ns	0.91
