# Supplementary material for: The adenovirus E4orf1 protein initiates a feedback loop involving insulin and growth factor receptors, AKT, and NF-κB, leading to abnormal DNA content in infected cells
Source: PLoS Pathog. 2025 Oct 27;21(10):e1013202. doi: 10.1371/journal.ppat.1013202 (PMC12578350; doi:10.1371/journal.ppat.1013202)
Supplement: S6 Fig — The P values were calculated using a two-way analysis of variance (ANOVA) with Holm–Šídák’s multiple comparisons test. (PDF) [file ppat.1013202.s006.pdf]

Summary stats for Fig 4A comparing the effects of AG1024 and AG1478 on the cell cycle in mock and Ad-infected A549 cells in 10% FBS.

Holm-Šídák's multiple comparisons test	<G1		G1		S		G2/M		DNA>4n	
n=3-6	Summary	Adjusted P	Summary	Adjusted P	Summary	Adjusted P	Summary	Adjusted P	Summary	Adjusted P
Mock vs. Mock + AG1024	ns	>0.99	ns	>0.99	ns	>0.99	ns	>0.99	ns	0.91
Mock vs. Mock + AG1478	ns	>0.99	ns	>0.99	ns	0.52	ns	>0.99	ns	0.83
Mock vs. ΔE1B	ns	>0.99	***	<0.001	ns	>0.99	ns	>0.99	***	<0.001
Mock vs. ΔE1B + AG1024	ns	>0.99	***	<0.001	ns	0.68	***	<0.001	***	<0.001
Mock vs. ΔE1B + AG1478	ns	>0.99	***	<0.001	ns	>0.99	***	<0.001	***	<0.001
Mock vs. ΔE4orf1	ns	>0.99	***	<0.001	ns	0.5	***	<0.001	***	<0.001
Mock vs. ΔE4orf1 + AG1024	ns	>0.99	***	<0.001	***	<0.001	***	<0.001	***	<0.001
Mock vs. ΔE4orf1 + AG1478	ns	>0.99	***	<0.001	***	<0.001	***	<0.001	***	<0.001
Mock + AG1024 vs. Mock + AG1478	ns	>0.99	ns	>0.99	ns	0.78	ns	0.92	ns	0.83
Mock + AG1024 vs. ΔE1B	ns	>0.99	***	<0.001	ns	>0.99	ns	>0.99	***	<0.001
Mock + AG1024 vs. ΔE1B + AG1024	ns	>0.99	***	<0.001	ns	0.86	***	<0.001	***	<0.001
Mock + AG1024 vs. ΔE1B + AG1478	ns	>0.99	***	<0.001	ns	>0.99	***	<0.001	***	<0.001
Mock + AG1024 vs. ΔE4orf1	ns	>0.99	***	<0.001	ns	0.78	***	<0.001	***	<0.001
Mock + AG1024 vs. ΔE4orf1 + AG1024	ns	>0.99	***	<0.001	***	<0.001	***	<0.001	***	<0.001
Mock + AG1024 vs. ΔE4orf1 + AG1478	ns	>0.99	***	<0.001	***	<0.001	***	<0.001	***	<0.001
Mock + AG1478 vs. ΔE1B	ns	>0.99	***	<0.001	ns	0.17	ns	>0.99	***	<0.001
Mock + AG1478 vs. ΔE1B + AG1024	ns	>0.99	***	<0.001	ns	>0.99	***	<0.001	***	<0.001
Mock + AG1478 vs. ΔE1B + AG1478	ns	>0.99	***	<0.001	ns	0.78	***	<0.001	***	<0.001
Mock + AG1478 vs. ΔE4orf1	ns	>0.99	***	<0.001	ns	>0.99	***	<0.001	***	<0.001
Mock + AG1478 vs. ΔE4orf1 + AG1024	ns	>0.99	***	<0.001	*	0.03	***	<0.001	***	<0.001
Mock + AG1478 vs. ΔE4orf1 + AG1478	ns	>0.99	***	<0.001	***	<0.001	***	<0.001	***	<0.001
ΔE1B vs. ΔE1B + AG1024	ns	>0.99	**	0.001	ns	0.2	***	<0.001	***	<0.001
ΔE1B vs. ΔE1B + AG1478	ns	>0.99	ns	>0.99	ns	0.88	***	<0.001	***	<0.001
ΔE1B vs. ΔE4orf1	ns	>0.99	ns	>0.99	ns	0.12	***	<0.001	***	<0.001
ΔE1B vs. ΔE4orf1 + AG1024	ns	>0.99	**	0.002	***	<0.001	***	<0.001	***	<0.001
ΔE1B vs. ΔE4orf1 + AG1478	ns	>0.99	ns	0.09	***	<0.001	***	<0.001	***	<0.001
ΔE1B + AG1024 vs. ΔE1B + AG1478	ns	>0.99	***	<0.001	ns	0.86	ns	0.07	ns	0.24
ΔE1B + AG1024 vs. ΔE4orf1	ns	>0.99	**	0.002	ns	>0.99	***	<0.001	*	0.03
ΔE1B + AG1024 vs. ΔE4orf1 + AG1024	ns	>0.99	ns	>0.99	***	<0.001	***	<0.001	***	<0.001
ΔE1B + AG1024 vs. ΔE4orf1 + AG1478	ns	>0.99	ns	0.88	***	<0.001	***	<0.001	***	<0.001
ΔE1B + AG1478 vs. ΔE4orf1	ns	>0.99	ns	>0.99	ns	0.78	**	0.002	***	<0.001
ΔE1B + AG1478 vs. ΔE4orf1 + AG1024	ns	>0.99	***	<0.001	***	<0.001	***	<0.001	***	<0.001
ΔE1B + AG1478 vs. ΔE4orf1 + AG1478	ns	>0.99	*	0.05	***	<0.001	***	<0.001	***	<0.001
ΔE4orf1 vs. ΔE4orf1 + AG1024	ns	>0.99	**	0.003	**	0.003	ns	>0.99	***	<0.001
ΔE4orf1 vs. ΔE4orf1 + AG1478	ns	>0.99	ns	0.13	***	<0.001	ns	>0.99	***	<0.001
ΔE4orf1 + AG1024 vs. ΔE4orf1 + AG147	ns	>0.99	ns	0.88	*	0.04	ns	>0.99	ns	0.24

Summary stats for Fig 4B comparing the effects of AG1024 and AG1478 on the cell cycle in mock and Ad-infected A549 cells in 1% FBS.

Holm-Šídák's multiple comparisons test	<G1		G1		S		G2/M		DNA>4n	
n=5-9	Summary	Adjusted P	Summary	Adjusted P	Summary	Adjusted P	Summary	Adjusted P	Summary	Adjusted P
Mock vs. Mock + AG1024	ns	0.9	ns	0.06	ns	0.99	ns	>0.99	ns	0.96
Mock vs. Mock + AG1478	ns	0.97	ns	>0.99	ns	0.99	ns	>0.99	ns	0.96
Mock vs. ΔE1B	ns	>0.99	***	<0.001	ns	0.97	ns	0.08	***	<0.001
Mock vs. ΔE1B + AG1024	ns	>0.99	***	<0.001	***	<0.001	ns	>0.99	ns	0.2
Mock vs. ΔE1B + AG1478	ns	>0.99	***	<0.001	***	<0.001	***	<0.001	***	<0.001
Mock vs. ΔE4orf1	ns	>0.99	***	<0.001	***	<0.001	***	<0.001	***	<0.001
Mock vs. ΔE4orf1 + AG1024	ns	>0.99	***	<0.001	***	<0.001	ns	>0.99	ns	0.68
Mock vs. ΔE4orf1 + AG1478	ns	>0.99	***	<0.001	***	<0.001	ns	>0.99	**	0.009
Mock + AG1024 vs. Mock + AG1478	ns	>0.99	ns	0.05	ns	0.96	ns	>0.99	ns	0.93
Mock + AG1024 vs. ΔE1B	ns	0.57	***	<0.001	ns	0.99	ns	0.08	***	<0.001
Mock + AG1024 vs. ΔE1B + AG1024	ns	0.96	***	<0.001	***	<0.001	ns	>0.99	ns	0.2
Mock + AG1024 vs. ΔE1B + AG1478	ns	0.74	***	<0.001	***	<0.001	***	<0.001	***	<0.001
Mock + AG1024 vs. ΔE4orf1	ns	0.6	***	<0.001	***	<0.001	***	<0.001	***	<0.001
Mock + AG1024 vs. ΔE4orf1 + AG1024	ns	0.98	***	<0.001	***	<0.001	ns	>0.99	ns	0.73
Mock + AG1024 vs. ΔE4orf1 + AG1478	ns	0.98	***	<0.001	***	<0.001	ns	>0.99	**	0.007
Mock + AG1478 vs. ΔE1B	ns	0.78	***	<0.001	ns	0.63	**	0.003	***	<0.001
Mock + AG1478 vs. ΔE1B + AG1024	ns	>0.99	***	<0.001	***	<0.001	ns	>0.99	ns	0.07
Mock + AG1478 vs. ΔE1B + AG1478	ns	0.9	***	<0.001	***	<0.001	***	<0.001	***	<0.001
Mock + AG1478 vs. ΔE4orf1	ns	0.8	***	<0.001	***	<0.001	***	<0.001	***	<0.001
Mock + AG1478 vs. ΔE4orf1 + AG1024	ns	>0.99	***	<0.001	***	<0.001	ns	0.98	ns	0.44
Mock + AG1478 vs. ΔE4orf1 + AG1478	ns	>0.99	***	<0.001	***	<0.001	ns	0.97	**	0.002
ΔE1B vs. ΔE1B + AG1024	ns	>0.99	***	<0.001	***	<0.001	*	0.03	***	<0.001
ΔE1B vs. ΔE1B + AG1478	ns	>0.99	ns	0.94	***	<0.001	ns	0.08	***	<0.001
ΔE1B vs. ΔE4orf1	ns	>0.99	ns	0.94	***	<0.001	ns	0.12	***	<0.001
ΔE1B vs. ΔE4orf1 + AG1024	ns	>0.99	***	<0.001	**	0.002	ns	0.12	***	<0.001
ΔE1B vs. ΔE4orf1 + AG1478	ns	>0.99	ns	0.22	***	<0.001	ns	0.11	***	<0.001
ΔE1B + AG1024 vs. ΔE1B + AG1478	ns	>0.99	***	<0.001	ns	0.99	***	<0.001	ns	0.07
ΔE1B + AG1024 vs. ΔE4orf1	ns	>0.99	***	<0.001	ns	0.99	***	<0.001	**	0.001
ΔE1B + AG1024 vs. ΔE4orf1 + AG1024	ns	>0.99	*	0.02	ns	0.07	ns	>0.99	ns	0.87
ΔE1B + AG1024 vs. ΔE4orf1 + AG1478	ns	>0.99	***	<0.001	***	<0.001	ns	>0.99	ns	0.73
ΔE1B + AG1478 vs. ΔE4orf1	ns	>0.99	ns	>0.99	ns	0.99	ns	>0.99	ns	0.73
ΔE1B + AG1478 vs. ΔE4orf1 + AG1024	ns	>0.99	***	<0.001	*	0.04	***	<0.001	**	0.009
ΔE1B + AG1478 vs. ΔE4orf1 + AG1478	ns	>0.99	ns	0.58	***	<0.001	***	<0.001	ns	0.73
ΔE4orf1 vs. ΔE4orf1 + AG1024	ns	>0.99	***	<0.001	ns	0.15	***	<0.001	***	<0.001
ΔE4orf1 vs. ΔE4orf1 + AG1478	ns	>0.99	ns	0.58	***	<0.001	***	<0.001	ns	0.12
ΔE4orf1 + AG1024 vs. ΔE4orf1 + AG147	ns	>0.99	***	<0.001	***	<0.001	ns	>0.99	ns	0.34
